# Structure of the mercury(II) mixed-halide (Br/Cl) complex of 2,2′-(5-*tert*-butyl-1,3-phenyl­ene)bis­(1-pentyl-1*H*-benzo[*d*]imidazole)

**DOI:** 10.1107/S2056989017002183

**Published:** 2017-02-21

**Authors:** Varsha Rani, Harkesh B. Singh, Ray J. Butcher

**Affiliations:** a Department of Chemistry, Indian Institute of Technology Bombay, Powai, Mumbai 400 076, India; b Department of Chemistry, Howard University, 525 College Street NW, Washington, DC 20059, USA

**Keywords:** crystal structure, single-crystal X-ray study, disorder, mercury coordination polymer, (benzimidazol-2-yl)benzene ligands

## Abstract

The mercury(II) complex of 2,2′-(5-*tert*-butyl-1,3-phenyl­ene)bis­(1-pentyl-1*H*-benz­imidazole), namely *catena*-poly[[dihalogenido­mercury(II)]-μ-2,2′-(5-*tert*-butyl-1,3-phenyl­ene)bis­(1-pentyl-1*H*-benzimidazole)-κ^2^ *N* ^3^:*N* ^3′^], [HgBr_1.52_Cl_0.48_(C_34_H_42_N_4_)], **2**, has a polymeric structure bridging *via* the N atoms from the benzimidazole moieties of the ligand. The compound crystallizes in the ortho­rhom­bic space group *Pca*2_1_ and is a racemic twin [BASF = 0.402 (9)]. The geometry around the Hg^II^ atom is distorted tetra­hedral, with the Hg^II^ atom coordinated to two N atoms, one Br atom, and a fourth coordination site is occupied by a mixed halide (Br/Cl). For the two ligands in the asymmetric unit, there is disorder with one of the two *tert*-butyl groups and benzimidazole moieties showing twofold disorder, with occupancy factors of 0.57 (2):0.43 (2) for the *tert*-butyl group and 0.73 (3):0.27 (3) for the benzimidazole group. In addition, there is threefold disorder for two of the four *n*-pentyl groups, with occupancy factors of 0.669 (4):0.177 (4):0.154 (4) and 0.662 (4):0.224 (4):0.154 (4), respectively. The mol­ecules form a one-dimensional helical polymer propagating in the *b*-axis direction. The helices are held together by intra-strand C—H⋯Br and C—H⋯Cl inter­actions. Each strand is further linked by inter-strand C—H⋯Br and C—H⋯Cl inter­actions. In addition, there are weak C—H⋯N inter-strand inter­actions which further stabilize the structural arrangement.

## Abstract 2

The structure of the mixed halide (Br/Cl) mercury complex of 2,2′-(5-*tert*-butyl-1,3-phenyl­ene)bis­(1-pentyl-1*H*-benzimidazole) is a one-dimensional helical polymer.

## Chemical context   

During the past few years, metallated complexes of 1,3-bis­(1*H*-benzimidazol-2-yl)benzene ligand have been well explored. This ligand is an ideal candidate for metalation due to the presence of two N atoms and one C atom which bind tightly with metal atoms (Carina *et al.*, 1997 ▸; Obara *et al.*, 2006 ▸; Karlsson *et al.*, 2011 ▸; Yang *et al.*, 2012 ▸; Tam *et al.*, 2012 ▸; Gonzalez, 2014 ▸). A highly phospho­rescent iridium complex has been reported with the bis­(benzimidazol-2-yl)benzene ligand (Obara *et al.*, 2006 ▸). Helical and nonhelical copper(I) complexes with bis­(benzimidazol-2-yl)benzene have been reported (Rüttimann *et al.*, 1992 ▸). A trimeric complex has been obtained through the self assembly of cyclo­metalated trinuclear palladium(II) complexes (Rüttimann *et al.*, 1993 ▸). Dinuclear zinc complexes containing (benzimidazol-2-yl)ben­zene based ligands have shown anti­cancer activities (Xie *et al.*, 2014 ▸).

A literature survey of mercury halide complexes with benzimidazole derivatives has shown that they come in two main types: polymeric, bridging either through the halide (Zhang *et al.*, 2015 ▸; Li *et al.*, 2007 ▸; Shen *et al.*, 2005 ▸) or through alternative N atoms from the benzimidazole moieties (Xiao *et al.*, 2009 ▸, 2011 ▸; Huang *et al.*, 2006 ▸; Li *et al.*, 2007 ▸, 2012*a* ▸,*b* ▸; Dey *et al.*, 2013 ▸; Du *et al.*, 2011 ▸; Chen *et al.*, 2013 ▸; Su *et al.*, 2003 ▸; Xu *et al.*, 2011 ▸), or as discrete mol­ecules (*i.e.* non-polymeric). A structurally related complex has been presented recently (Rani *et al.*, 2017 ▸).
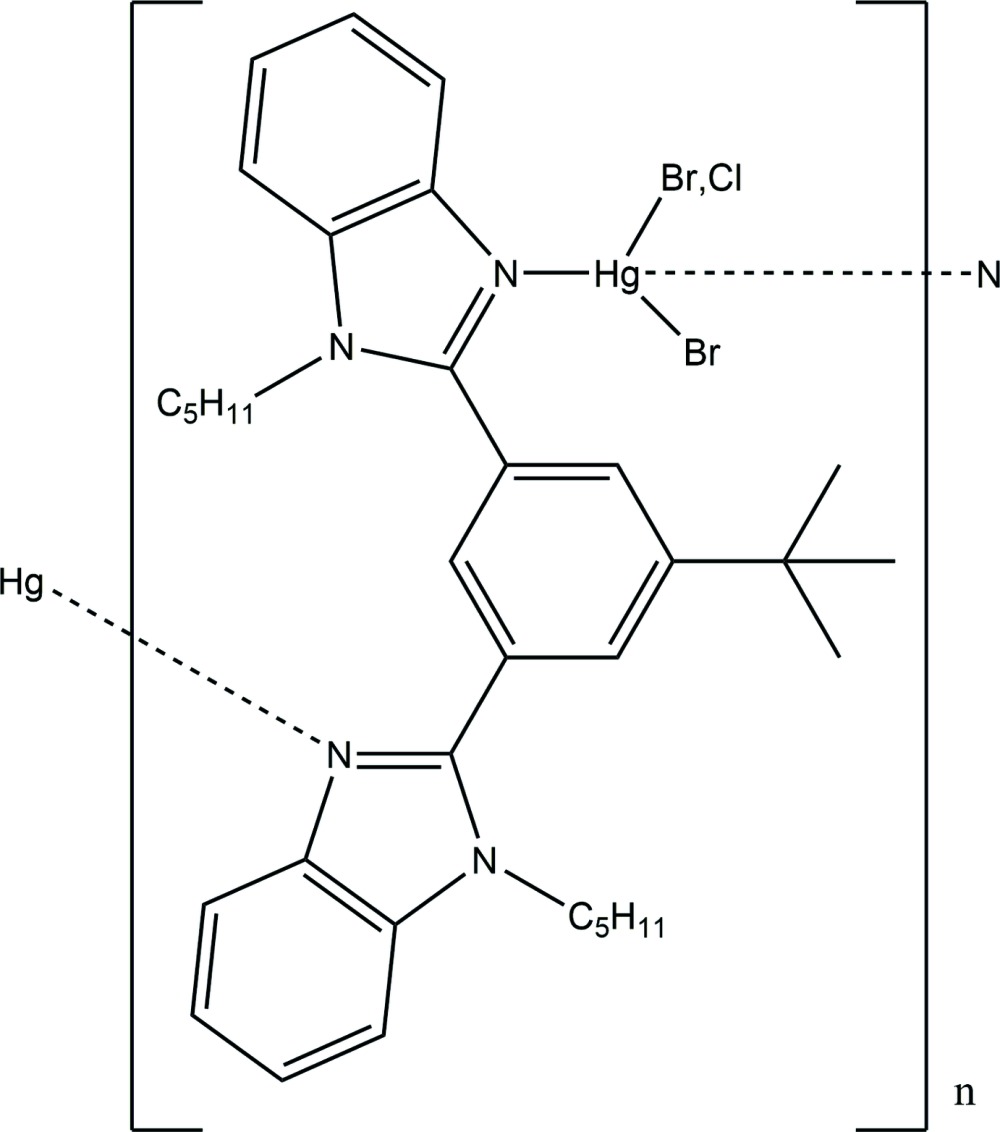


**Figure 1:**
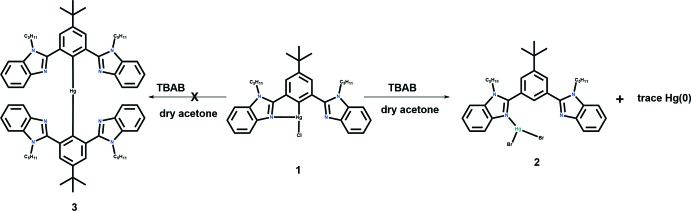
Diagram showing the starting compound, **1**, the title compound, **2**, and the expected product, **3**.

In an attempt to synthesize bis­[4-*tert*-butyl-2,6-bis­(1-pentyl-1*H*-benzimidazol-2-yl)phen­yl]mercury (**3**) from [4-*tert*-butyl-2,6-bis­(1-pentyl-1*H*-benzimidazol-2-yl)phen­yl]mercury(II) chlor­ide (C_34_H_41_N_4_HgCl), **1**, and tetra-*n*-butyl­ammonium bromide (Bu_4_N^+^Cl^−^) in dry acetone, the mercury complex of 2,2′-(5-*tert*-butyl-1,3-phenyl­ene)bis­(1-pentyl-1*H*-benzimidazole) [HgBr_1.52_Cl_0.48_(C_34_H_42_N_4_)], **2**, was isolated unexpectedly (Fig. 1 ▸). The procedure for the synthesis of complex **1** will be reported elsewhere.

## Structural commentary   

The title compound crystallizes in the ortho­rhom­bic space group *Pca*2_1_ and is a racemic twin [BASF = 0.402 (9)]. The geometry around the Hg^II^ atom is distorted tetra­hedral with the Hg^II^ atom coordinated to two N atoms, one Br atom, and a fourth coordination site is occupied by a mixed halide (Br/Cl). There are two such sites in the asymmetric unit and in one site, the Br/Cl occupancy is 0.599 (9):0.401 (9) and in the other site the ratio is 0.431 (9):0.569 (9), giving an overall composition (*L*)_2_HgBr_1.52_Cl_0.48_, where *L* is 2,2′-(5-*tert*-butyl-1,3-phenyl­ene)bis­(1-pentyl-1*H*-benzimidazole). Unlike the case with the recently published structure (Rani *et al.*, 2017 ▸) of a related ligand where both coordinating positions are blocked by substituents (Br and *tert*-but­yl), even though in the present ligand one position is open for the formation of an Hg—C bond, the basic arrangement is the same as in the previous structure.

**Figure 2:**
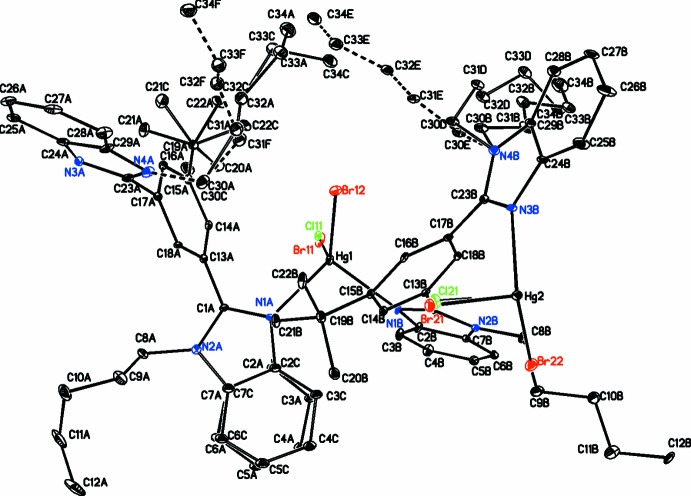
Diagram showing the contents of the asymmetric unit and the disorder in the various moieties. Atomic displacement parameters are shown at the 30% probability level.

For the two ligands in the asymmetric unit, there is considerable disorder, with one of the two *tert*-butyl groups and benzimidazole moieties showing twofold disorder, with occupancy factors of 0.57 (2):0.43 (2) for the *tert*-butyl group and 0.73 (3):0.27 (3) for the benzimidazole group. In addition, there is threefold disorder for two of the four *n*-pentyl groups, with occupancy factors of 0.669 (4):0.177 (4):0.154 (4) and 0.662 (4):0.224 (4):0.154 (4), respectively, as shown in Fig. 2 ▸. The two sets of Hg—N bond lengths are 2.313 (7)/2.343 (6) and 2.316 (6)/2.327 (6) Å, which span a wider range than that observed (Rani *et al.*, 2017 ▸) in a structurally similar complex [2.333 (4)–2.338 (4) Å]. The two sets of Hg—Cl and Hg—Br bond lengths are 2.37 (2)/2.48 (4) (the larger s.u. values are due to the disorder of the anions) and 2.508 (1)/2.593 (4) and 2.515 (1)/2.57 (2) Å, respectively. As mentioned above, the coordination environments are distorted tetra­hedral for both Hg^II^ atoms, with bond angles ranging from 97.2 (2) to 124.1 (7)°, values that are more distorted than those found in the analogous complex [100.6 (1)–126.35 (7)°] due to the disorder in the anions.

**Figure 3:**
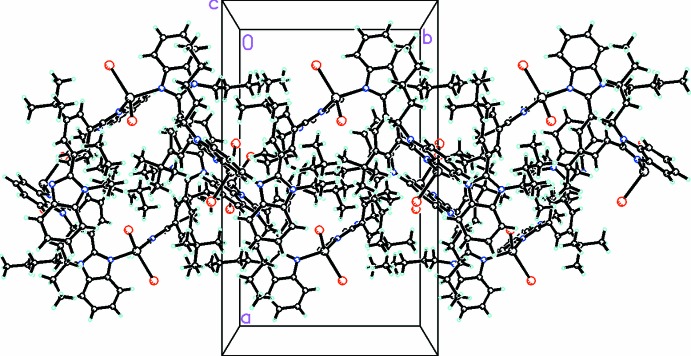
Diagram containing two units of the polymer illustrating its zigzag nature, with H atoms omitted for clarity (symmetry code for generating the second unit: *x*, *y* − 1, *z*). Minor components of both the pentyl and benzimidazole disorder, as well as the chloride-anion disorder, have been omitted for clarity. H atoms have also been omitted for clarity. Atomic displacement parameters are shown at the 30% probability level.

The discussion of the conformations adopted by the ligand will be restricted to just those of the major components for the disordered moieties where the ligand has adopted a conformation where the two sets of two benzimidazole moieties are not coplanar with each other or their central arene ring. The dihedral angles between the two sets of benzimidazole moieties are 76.4 (3) and 88.0 (2)° (Table 1 ▸), while their dihedral angles with the central ring are 60.1 (3)/59.5 (3) and 50.1 (3)/60.2 (2)°. These values are significantly different from those observed in the related complex (Rani *et al.*, 2017 ▸), where the corresponding angles are 54.9 (6) and 65.6 (4)° between the two benzimidazole moieties (two values due to the disorder of one of these moieties) and 55.6 (1) and 89.3 (5)/79.2 (5)° with the central ring. Further, the four pentyl side chains have adopted quite different conformations. This can be seen by examining the dihedral angles of the C atoms in each of these groups. There are four such angles for each, C(from the planar benzimidazole ring)—N—C—C, N—C—C—C, C—C—C—C, and C—C—C—C (last C is a terminal CH_3_ group). The four sets of values are −115.6 (11)/−166.7 (11)/76.2 (19)/67.5 (19), 102.2 (12)/−61.5 (15)/−171.9 (13)/173.0 (16)°, 113.4 (10)/162.5 (9)/−174.7 (10)/−179.5 (11) and −104.2 (15)/−172.4 (13)/−78.2 (15)/177.1 (17)°. From these values it can be seen that the first two pentyl groups have adopted a slightly curled-up conformation, while the last two are in a fully extended all-*trans* conformation (Fig. 3 ▸).

## Supra­molecular features   

**Figure 4:**
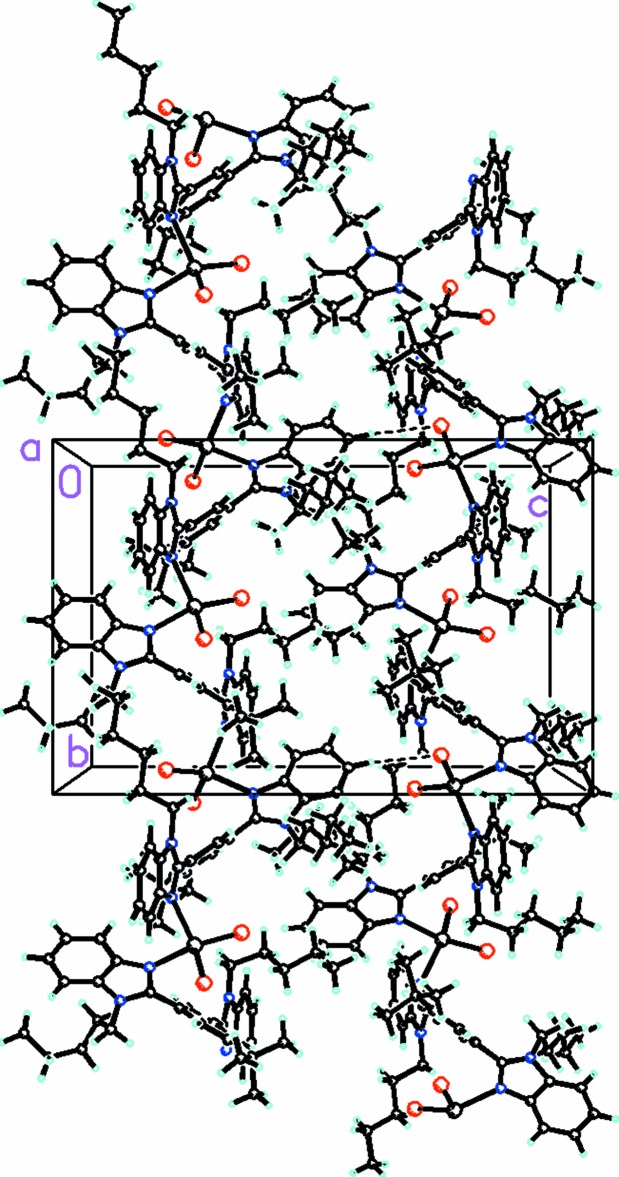
Packing diagram, viewed along the *c* axis, showing two strands of the polymer which propagate in the *b*-axis direction.

The mol­ecules form a one-dimensional helical polymer pro­pagating in the *b* direction (Fig. 4 ▸). The helices are held together by intra-strand C—H⋯Br and C—H⋯Cl inter­actions. Each strand is further linked by inter-strand C—H⋯Br and C—H⋯Cl inter­actions. There are inter-strand π–π inter­actions between the benzimidazole moieties which help stabilize the packing [N1*B*/N2*B*/C1*B*/C2*B*/C7*B*⋯C24*A*–C29*A* (symmetry code: 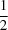 + *x*, 1 − *y*, *z*) and N3*A*/N4*A*/C23*A*/C24*A*/C29*A*⋯C2*B*–C7*B* (symmetry code: *x* − 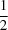, 1 − *y*, *z*]. In addition, there are weak C—H⋯N inter­actions which further stabilize the structural arrangement. Numerical details are given in Table 2 ▸.

## Database survey   

A survey of the structural investigations of mercury halide complexes with benzimidazole derivatives has shown that they come in two main types; polymeric, bridging either through the halide (Zhang *et al.*, 2015 ▸; Li *et al.*, 2007 ▸; Shen *et al.*, 2005 ▸) or through alternative N atoms from the benzimidazole moieties (Xiao *et al.*, 2009 ▸, 2011 ▸; Huang *et al.*, 2006 ▸; Li *et al.*, 2007 ▸, 2012*a* ▸,*b* ▸; Dey *et al.*, 2013 ▸; Du *et al.*, 2011 ▸; Chen *et al.*, 2013 ▸; Su *et al.*, 2003 ▸; Xu *et al.*, 2011 ▸), or discrete mol­ecules, *i.e.* nonpolymeric (Xiao *et al.*, 2011 ▸; Wu *et al.*, 2009 ▸; Zhao *et al.*, 2012 ▸; Lou *et al.*, 2012 ▸; Zhu *et al.*, 2009 ▸; Carballo *et al.*, 1993 ▸; Yan *et al.*, 2012 ▸; Hu *et al.*, 2012 ▸, 2015 ▸; Ding *et al.*, 2012 ▸; Matthews *et al.*, 1998 ▸; Manjunatha *et al.*, 2011 ▸; Wang *et al.*, 2007 ▸, 2009 ▸, 2012 ▸, 2015 ▸; Chen *et al.*, 2014 ▸; Su *et al.*, 2003 ▸; Quiroz-Castro *et al.*, 2000 ▸; Yang & Luo, 2012 ▸; He *et al.*, 2012 ▸; Bouchouit *et al.*, 2015 ▸).

## Synthesis and crystallization   

To a solution of **1** (0.5 g, 0.67 mmol) in dry acetone was added tetra-*n*-butyl­ammonium bromide (0.26 g, 0.82 mmol) at room temperature. The reaction mixture was refluxed for 3 h under an inert atmosphere of N_2_ and then filtered through Whatman filter paper. The residue contained shiny mercury particles which could be witnessed with the naked eye and the solvent was evaporated from the filtrate and washed with petroleum ether and then with diethyl ether. The compound was dried under *vacuo*. Colorless plate-shaped single crystals were obtained from DMSO at 291 K (yield 18%, 0.21 g). ^1^H NMR (500 MHz, DMSO): δ 7.96–7.93 (*m*, 3H), 7.74 (*d*, *J* = 7.9 Hz, 2H), 7.70 (*d*, *J* = 7.9 Hz, 2H), 7.33–7.26 (*m*, 4H), 4.35 (*t*, *J* = 7.4 Hz, 4H), 1.72 (*m*, 4H), 1.42 (*s*, 9 H), 1.15–0.93 (*m*, 8H), 0.71 (*t*, *J* = 6.8 Hz, 6H). Analysis calculated for C_34_H_42_Br_1.52_Cl_0.48_HgN_4_: C 48.33, H 5.01, N 6.63%; found: C 49.73, H 5.14, N 6.84%.

## Refinement   

Crystal data, data collection and structure refinement details are summarized in Table 3 ▸. The title compound was refined as a racemic twin [BASF = 0.402 (9)]. The H atoms were positioned geometrically and refined as riding, with C—H = 0.95–0.98 Å and *U_iso_*(H) = 1.5*U*_eq_(C) for methyl H atoms and 1.2*U*_eq_(C) otherwise. For the two ligands in the asymmetric unit, there is considerable disorder with one of the two *tert*-butyl groups and benzimidazole moieties showing twofold disorder, with occupancy factors of 0.57 (2):0.43 (2) for the *tert*-butyl group and 0.73 (3):0.27 (3) for the benzimidazole group. The displacement parameters of the disordered *tert*-butyl group were restrained to be similar (SIMU command). One C atom (C22*C*) from the minor component was additionally restrained to be close to isotropic (ISOR 0.005 command). The two components of the benzimidazole moiety were restrained to be planar using the FLAT command and their metrical and displacement parameters were restrained using the SIMU and SADI commands in *SHELXL2016* (Sheldrick, 2015 ▸). In addition, there is threefold disorder (occupancies summed to unity using the SUMP command) for two of the four *n*-pentyl groups, with occupancy factors of 0.669 (4):0.177 (4):0.154 (4) and 0.662 (4):0.224 (4):0.154 (4), respectively. For one pentyl group, some atoms (C30*A*–C32*A* and C30*C*–C32*C*) of two of the three components were refined as overlapping (using EXYZ commands). For the other disordered pentyl group, all atoms of all three components adopt independent conformations. One of the occupancy rates (of the least prevalent moieties *E* and *F*) is shared between the two disordered sites due to mutually exclusive overlap of atoms.

## Supplementary Material

Crystal structure: contains datablock(s) I. DOI: 10.1107/S2056989017002183/zl2695sup1.cif

Structure factors: contains datablock(s) I. DOI: 10.1107/S2056989017002183/zl2695Isup2.hkl

CCDC reference: 1531940

Additional supporting information:  crystallographic information; 3D view; checkCIF report

## Figures and Tables

**Table 1:** Selected geometric parameters (Å, °)

Hg1—N1*A*	2.313 (7)	Hg2—N3*A* ^i^	2.316 (6)
Hg1—N1*B*	2.343 (6)	Hg2—N3*B*	2.327 (6)
Hg1—Cl11	2.373 (19)	Hg2—Cl21	2.48 (4)
Hg1—Br12	2.5083 (10)	Hg2—Br22	2.5147 (11)
Hg1—Br11	2.593 (4)	Hg2—Br21	2.57 (2)
			
N1*A*—Hg1—N1*B*	99.4 (3)	N3*A* ^i^—Hg2—N3*B*	97.2 (2)
N1*A*—Hg1—Cl11	107.1 (6)	N3*A* ^i^—Hg2—Cl21	101.7 (8)
N1*B*—Hg1—Cl11	108.0 (5)	N3*B*—Hg2—Cl21	105.0 (9)
N1*A*—Hg1—Br12	115.82 (18)	N3*A* ^i^—Hg2—Br22	115.13 (18)
N1*B*—Hg1—Br12	111.26 (17)	N3*B*—Hg2—Br22	109.95 (17)
Cl11—Hg1—Br12	114.1 (5)	Cl21—Hg2—Br22	124.1 (7)
N1*A*—Hg1—Br11	103.9 (2)	N3*A* ^i^—Hg2—Br21	105.3 (5)
N1*B*—Hg1—Br11	105.2 (2)	N3*B*—Hg2—Br21	107.8 (5)
Br12—Hg1—Br11	119.01 (12)	Br22—Hg2—Br21	119.1 (4)

Symmetry code: (i) 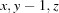.

**Table 2:** Hydrogen-bond geometry (Å, °)

*D*—H⋯*A*	*D*—H	H⋯*A*	*D*⋯*A*	*D*—H⋯*A*
C3*A*—H3*AA*⋯N1*B*	0.95	2.45	3.29 (4)	148
C8*A*—H8*AB*⋯Br22^ii^	0.99	2.94	3.873 (11)	158
C25*A*—H25*A*⋯Br21^ii^	0.95	2.96	3.66 (2)	131
C25*A*—H25*A*⋯Cl21^ii^	0.95	2.74	3.45 (3)	132
C28*A*—H28*A*⋯Br11^iii^	0.95	2.96	3.793 (12)	147
C28*A*—H28*A*⋯Cl11^iii^	0.95	2.86	3.70 (2)	149
C30*A*—H30*B*⋯Br11^iii^	0.99	3.04	3.979 (13)	158
C3*B*—H3*BA*⋯Br11	0.95	2.96	3.631 (11)	129
C6*B*—H6*BA*⋯Br21^iv^	0.95	2.88	3.75 (2)	153
C6*B*—H6*BA*⋯Cl21^iv^	0.95	2.89	3.73 (4)	149
C25*B*—H25*B*⋯N3*A* ^i^	0.95	2.56	3.332 (14)	139
C30*D*—H30*G*⋯Br12	0.99	3.06	3.95 (5)	150

Symmetry codes: (i) 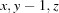; (ii) 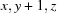; (iii) 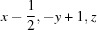; (iv) 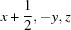.

**Table 3:** Experimental details

Crystal data	
Chemical formula	[HgBr_1.52_Cl_0.48_(C_34_H_42_N_4_)]
*M*_r_	845.56
Crystal system, space group	Orthorhombic, *P* *c* *a*2_1_
Temperature (K)	100
*a*, *b*, *c* (Å)	22.8887 (14), 13.8830 (8), 21.1396 (12)
*V* (Å^3^)	6717.4 (7)
*Z*	8
Radiation type	Cu *K*α
μ (mm^−1^)	10.90
Crystal size (mm)	0.19 × 0.17 × 0.07

Data collection	
Diffractometer	Bruker APEXII CCD
Absorption correction	Multi-scan (*SADABS*; Sheldrick, 1996 ▸)
*T*_min_, *T*_max_	0.394, 0.753
No. of measured, independent and observed [*I* > 2σ(*I*)] reflections	93252, 11762, 11431
*R*_int_	0.043
(sin θ/λ)_max_ (Å^−1^)	0.596

Refinement	
*R*[*F* ^2^ > 2σ(*F* ^2^)], *wR*(*F* ^2^), *S*	0.029, 0.069, 1.14
No. of reflections	11762
No. of parameters	997
No. of restraints	888
H-atom treatment	H-atom parameters constrained
Δρ_max_, Δρ_min_ (e Å^−3^)	1.38, −0.85
Absolute structure	Refined as an inversion twin
Absolute structure parameter	0.402 (9)

Computer programs: *APEX2* (Bruker, 2005 ▸), *SAINT* (Bruker, 2002 ▸), *SUPERFLIP* (Palatinus & Chapuis, 2007 ▸), *SHELXL2016* (Sheldrick, 2015 ▸) and *SHELXTL* (Sheldrick, 2008 ▸).

## supplementary crystallographic information

### Crystal data

**Table:**

[HgBr_1.52_Cl_0.48_(C_34_H_42_N_4_)]	*D*_x_ = 1.672 Mg m^−^^3^
*M**_r_* = 845.56	Cu *K*α radiation, λ = 1.54184 Å
Orthorhombic, *P**c**a*2_1_	Cell parameters from 9060 reflections
*a* = 22.8887 (14) Å	θ = 3.7–66.7°
*b* = 13.8830 (8) Å	µ = 10.90 mm^−^^1^
*c* = 21.1396 (12) Å	*T* = 100 K
*V* = 6717.4 (7) Å^3^	Plate, colourless
*Z* = 8	0.19 × 0.17 × 0.07 mm
*F*(000) = 3322	

### Data collection

**Table:**

Bruker APEXII CCD diffractometer	11431 reflections with *I* > 2σ(*I*)
ω scans	*R*_int_ = 0.043
Absorption correction: multi-scan (SADABS; Sheldrick, 1996)	θ_max_ = 66.8°, θ_min_ = 3.7°
*T*_min_ = 0.394, *T*_max_ = 0.753	*h* = −27→26
93252 measured reflections	*k* = −16→16
11762 independent reflections	*l* = −25→24

### Refinement

**Table:**

Refinement on *F*^2^	Hydrogen site location: inferred from neighbouring sites
Least-squares matrix: full	H-atom parameters constrained
*R*[*F*^2^ > 2σ(*F*^2^)] = 0.029	*w* = 1/[σ^2^(*F*_o_^2^) + (0.0061*P*)^2^ + 31.5501*P*] where *P* = (*F*_o_^2^ + 2*F*_c_^2^)/3
*wR*(*F*^2^) = 0.069	(Δ/σ)_max_ = 0.005
*S* = 1.14	Δρ_max_ = 1.38 e Å^−^^3^
11762 reflections	Δρ_min_ = −0.85 e Å^−^^3^
997 parameters	Absolute structure: Refined as an inversion twin
888 restraints	Absolute structure parameter: 0.402 (9)

### Special details

**Table:**

Geometry. All esds (except the esd in the dihedral angle between two l.s. planes) are estimated using the full covariance matrix. The cell esds are taken into account individually in the estimation of esds in distances, angles and torsion angles; correlations between esds in cell parameters are only used when they are defined by crystal symmetry. An approximate (isotropic) treatment of cell esds is used for estimating esds involving l.s. planes.
Refinement. Refined as a 2-component inversion twin

### Fractional atomic coordinates and isotropic or equivalent isotropic displacement parameters (Å^2^)

**Table:**

	*x*	*y*	*z*	*U*_iso_*/*U*_eq_	Occ. (<1)
Hg1	0.73599 (2)	0.47092 (2)	0.25318 (2)	0.02440 (9)	
Hg2	0.51919 (2)	−0.02075 (2)	0.26809 (2)	0.02425 (9)	
Br12	0.66545 (5)	0.44471 (7)	0.34233 (5)	0.0370 (2)	
Br22	0.60239 (5)	−0.03762 (8)	0.19119 (5)	0.0445 (3)	
Br11	0.83121 (16)	0.5668 (4)	0.2745 (3)	0.0346 (9)	0.599 (9)
Br21	0.4296 (10)	0.0792 (15)	0.2364 (8)	0.0278 (19)	0.431 (9)
Cl11	0.8204 (7)	0.5600 (15)	0.2819 (12)	0.0346 (9)	0.401 (9)
Cl21	0.4287 (19)	0.072 (3)	0.2464 (15)	0.0278 (19)	0.569 (9)
N1A	0.6978 (3)	0.5423 (5)	0.1634 (3)	0.0244 (15)	
N2A	0.6527 (4)	0.6503 (6)	0.1029 (4)	0.0329 (17)	
N3A	0.4820 (3)	0.8355 (5)	0.3065 (3)	0.0212 (14)	
N4A	0.4668 (4)	0.6774 (6)	0.3121 (4)	0.034 (2)	
C1A	0.6660 (4)	0.6220 (6)	0.1630 (4)	0.0216 (17)	
C2A	0.7011 (14)	0.511 (3)	0.1012 (13)	0.032 (4)	0.27 (3)
C3A	0.7268 (19)	0.429 (3)	0.0766 (17)	0.036 (4)	0.27 (3)
H3AA	0.745915	0.382720	0.102702	0.044*	0.27 (3)
C4A	0.723 (2)	0.417 (3)	0.0106 (17)	0.042 (4)	0.27 (3)
H4AA	0.738836	0.361701	−0.009104	0.051*	0.27 (3)
C5A	0.696 (2)	0.486 (3)	−0.0251 (18)	0.043 (4)	0.27 (3)
H5AA	0.695603	0.476618	−0.069650	0.052*	0.27 (3)
C6A	0.6696 (19)	0.567 (3)	−0.002 (2)	0.039 (4)	0.27 (3)
H6AA	0.650217	0.611915	−0.028707	0.047*	0.27 (3)
C7A	0.673 (2)	0.579 (3)	0.064 (2)	0.035 (4)	0.27 (3)
C2C	0.7089 (6)	0.5217 (12)	0.0993 (7)	0.032 (2)	0.73 (3)
C3C	0.7434 (8)	0.4511 (14)	0.0700 (7)	0.036 (3)	0.73 (3)
H3CA	0.764338	0.405323	0.094426	0.043*	0.73 (3)
C4C	0.7461 (10)	0.4498 (13)	0.0038 (7)	0.043 (3)	0.73 (3)
H4CA	0.769263	0.402372	−0.016718	0.052*	0.73 (3)
C5C	0.7163 (9)	0.5152 (14)	−0.0319 (7)	0.044 (3)	0.73 (3)
H5CA	0.717806	0.510586	−0.076686	0.053*	0.73 (3)
C6C	0.6840 (8)	0.5876 (14)	−0.0047 (8)	0.040 (3)	0.73 (3)
H6CA	0.665240	0.635417	−0.029569	0.048*	0.73 (3)
C7C	0.6800 (8)	0.5878 (12)	0.0614 (8)	0.036 (3)	0.73 (3)
C8A	0.6258 (5)	0.7407 (8)	0.0829 (5)	0.043 (3)	
H8AA	0.651868	0.772989	0.052158	0.052*	
H8AB	0.621789	0.783556	0.120103	0.052*	
C9A	0.5670 (5)	0.7270 (11)	0.0533 (6)	0.058 (4)	
H9A1	0.538239	0.709685	0.086429	0.070*	
H9A2	0.568784	0.673280	0.022563	0.070*	
C10A	0.5470 (7)	0.8182 (15)	0.0197 (7)	0.089 (6)	
H10A	0.554632	0.874029	0.047668	0.107*	
H10B	0.504323	0.814408	0.012834	0.107*	
C11A	0.5770 (8)	0.8360 (17)	−0.0441 (8)	0.102 (8)	
H11A	0.566321	0.901378	−0.058928	0.122*	
H11B	0.619796	0.835147	−0.037520	0.122*	
C12A	0.5624 (7)	0.7661 (18)	−0.0949 (7)	0.103 (8)	
H12A	0.519933	0.758753	−0.097678	0.154*	
H12B	0.580235	0.703615	−0.085154	0.154*	
H12C	0.577564	0.789703	−0.135416	0.154*	
C13A	0.6456 (4)	0.6727 (6)	0.2197 (4)	0.0258 (19)	
C14A	0.6867 (4)	0.7111 (5)	0.2622 (4)	0.0249 (17)	
H14A	0.727217	0.701597	0.254546	0.030*	
C15A	0.6689 (4)	0.7630 (6)	0.3156 (4)	0.028 (2)	
C16A	0.6096 (4)	0.7737 (6)	0.3256 (4)	0.0258 (19)	
H16A	0.596773	0.809324	0.361339	0.031*	
C17A	0.5682 (4)	0.7346 (6)	0.2854 (4)	0.0223 (18)	
C18A	0.5862 (4)	0.6849 (6)	0.2315 (4)	0.0220 (17)	
H18A	0.558102	0.659552	0.202911	0.026*	
C19A	0.7129 (5)	0.8058 (8)	0.3625 (5)	0.035 (2)	
C20A	0.7774 (9)	0.794 (2)	0.3430 (11)	0.056 (5)	0.573 (19)
H20A	0.785422	0.834713	0.306063	0.084*	0.573 (19)
H20B	0.785065	0.726650	0.332412	0.084*	0.573 (19)
H20C	0.802608	0.813832	0.378150	0.084*	0.573 (19)
C21A	0.7029 (9)	0.9158 (13)	0.3683 (12)	0.046 (5)	0.573 (19)
H21A	0.732492	0.943457	0.396455	0.069*	0.573 (19)
H21B	0.663934	0.927903	0.385666	0.069*	0.573 (19)
H21C	0.706074	0.945532	0.326377	0.069*	0.573 (19)
C22A	0.7053 (12)	0.763 (2)	0.4258 (10)	0.063 (6)	0.573 (19)
H22A	0.710394	0.693303	0.423325	0.094*	0.573 (19)
H22B	0.666024	0.777947	0.441571	0.094*	0.573 (19)
H22C	0.734459	0.790368	0.454767	0.094*	0.573 (19)
C20C	0.7617 (16)	0.848 (3)	0.3303 (15)	0.057 (6)	0.427 (19)
H20D	0.792848	0.861297	0.360873	0.085*	0.427 (19)
H20E	0.749512	0.907867	0.309942	0.085*	0.427 (19)
H20F	0.776155	0.802804	0.298194	0.085*	0.427 (19)
C21C	0.6807 (14)	0.870 (2)	0.4101 (16)	0.061 (7)	0.427 (19)
H21D	0.650612	0.832825	0.431885	0.092*	0.427 (19)
H21E	0.662311	0.924245	0.387547	0.092*	0.427 (19)
H21F	0.708620	0.895641	0.441063	0.092*	0.427 (19)
C22C	0.7342 (11)	0.717 (2)	0.4038 (12)	0.042 (5)	0.427 (19)
H22D	0.767265	0.737330	0.430250	0.062*	0.427 (19)
H22E	0.746442	0.664732	0.375940	0.062*	0.427 (19)
H22F	0.702144	0.695380	0.430944	0.062*	0.427 (19)
C23A	0.5062 (4)	0.7496 (6)	0.2999 (4)	0.0256 (19)	
C24A	0.4243 (4)	0.8198 (7)	0.3246 (4)	0.027 (2)	
C25A	0.3788 (4)	0.8842 (8)	0.3388 (4)	0.032 (2)	
H25A	0.384475	0.951959	0.337494	0.038*	
C26A	0.3256 (4)	0.8445 (8)	0.3546 (5)	0.036 (2)	
H26A	0.293990	0.885989	0.364851	0.044*	
C27A	0.3170 (5)	0.7458 (9)	0.3560 (5)	0.041 (3)	
H27A	0.279136	0.722255	0.366122	0.049*	
C28A	0.3599 (5)	0.6809 (8)	0.3436 (5)	0.040 (3)	
H28A	0.353422	0.613343	0.345219	0.048*	
C29A	0.4149 (5)	0.7210 (7)	0.3280 (5)	0.035 (2)	
C30A	0.4780 (6)	0.5738 (7)	0.3149 (5)	0.047 (2)	0.669 (4)
H30A	0.515077	0.559681	0.292699	0.057*	0.669 (4)
H30B	0.446264	0.539475	0.292500	0.057*	0.669 (4)
C31A	0.4820 (7)	0.5352 (9)	0.3840 (6)	0.048 (2)	0.669 (4)
H31A	0.444301	0.547779	0.405430	0.057*	0.669 (4)
H31B	0.487401	0.464457	0.382378	0.057*	0.669 (4)
C32A	0.5307 (6)	0.5780 (9)	0.4239 (6)	0.050 (2)	0.669 (4)
H32A	0.522451	0.647020	0.431602	0.060*	0.669 (4)
H32B	0.568025	0.573400	0.400524	0.060*	0.669 (4)
C33A	0.5368 (10)	0.5255 (14)	0.4880 (8)	0.054 (4)	0.669 (4)
H33A	0.501421	0.536933	0.513971	0.065*	0.669 (4)
H33B	0.540386	0.455297	0.480796	0.065*	0.669 (4)
C34A	0.5902 (9)	0.5620 (18)	0.5228 (10)	0.069 (5)	0.669 (4)
H34A	0.586961	0.631841	0.528797	0.103*	0.669 (4)
H34B	0.625269	0.547591	0.497989	0.103*	0.669 (4)
H34C	0.592885	0.530354	0.564120	0.103*	0.669 (4)
C30C	0.4780 (6)	0.5738 (7)	0.3149 (5)	0.047 (2)	0.177 (4)
H30E	0.515077	0.559681	0.292699	0.057*	0.177 (4)
H30F	0.446264	0.539475	0.292500	0.057*	0.177 (4)
C31C	0.4820 (7)	0.5352 (9)	0.3840 (6)	0.048 (2)	0.177 (4)
H31E	0.444301	0.547779	0.405430	0.057*	0.177 (4)
H31F	0.487401	0.464457	0.382378	0.057*	0.177 (4)
C32C	0.5307 (6)	0.5780 (9)	0.4239 (6)	0.050 (2)	0.177 (4)
H32E	0.522609	0.648012	0.425432	0.060*	0.177 (4)
H32F	0.566630	0.570469	0.398389	0.060*	0.177 (4)
C33C	0.548 (4)	0.550 (4)	0.4915 (17)	0.052 (4)	0.177 (4)
H33E	0.580660	0.591743	0.506019	0.062*	0.177 (4)
H33F	0.514593	0.559825	0.520391	0.062*	0.177 (4)
C34C	0.567 (3)	0.447 (4)	0.492 (3)	0.054 (8)	0.177 (4)
H34D	0.599136	0.437561	0.462772	0.080*	0.177 (4)
H34E	0.533746	0.406027	0.479784	0.080*	0.177 (4)
H34F	0.579278	0.429341	0.535156	0.080*	0.177 (4)
C30F	0.4780 (6)	0.5738 (7)	0.3149 (5)	0.047 (2)	0.154 (3)
H30K	0.499902	0.553477	0.276837	0.057*	0.154 (3)
H30L	0.440435	0.538498	0.315708	0.057*	0.154 (3)
C31F	0.513 (2)	0.550 (5)	0.3741 (15)	0.050 (3)	0.154 (3)
H31K	0.513851	0.478893	0.380099	0.060*	0.154 (3)
H31L	0.554043	0.571619	0.368411	0.060*	0.154 (3)
C32F	0.488 (2)	0.597 (5)	0.4327 (19)	0.051 (4)	0.154 (3)
H32K	0.482928	0.666949	0.425206	0.061*	0.154 (3)
H32L	0.514504	0.588202	0.468891	0.061*	0.154 (3)
C33F	0.428 (3)	0.552 (4)	0.448 (3)	0.051 (5)	0.154 (3)
H33K	0.402891	0.553032	0.410377	0.061*	0.154 (3)
H33L	0.433376	0.484577	0.461783	0.061*	0.154 (3)
C34F	0.399 (3)	0.608 (6)	0.501 (3)	0.057 (13)	0.154 (3)
H34P	0.425443	0.611499	0.537044	0.085*	0.154 (3)
H34Q	0.362853	0.575704	0.512842	0.085*	0.154 (3)
H34R	0.390325	0.673309	0.485689	0.085*	0.154 (3)
N1B	0.7667 (3)	0.3243 (5)	0.2096 (3)	0.0223 (15)	
N2B	0.7794 (3)	0.1645 (5)	0.2095 (3)	0.0224 (15)	
N3B	0.5535 (3)	0.0389 (5)	0.3640 (3)	0.0190 (14)	
N4B	0.6096 (3)	0.1219 (5)	0.4286 (4)	0.0270 (16)	
C1B	0.7416 (4)	0.2388 (6)	0.2209 (4)	0.0203 (17)	
C2B	0.8237 (4)	0.3043 (6)	0.1898 (5)	0.0248 (19)	
C3B	0.8688 (4)	0.3664 (7)	0.1718 (5)	0.034 (2)	
H3BA	0.863281	0.434172	0.170396	0.041*	
C4B	0.9215 (4)	0.3251 (8)	0.1564 (6)	0.038 (2)	
H4BA	0.953081	0.365729	0.144579	0.046*	
C5B	0.9302 (4)	0.2248 (7)	0.1574 (5)	0.031 (2)	
H5BA	0.967241	0.199101	0.146059	0.038*	
C6B	0.8858 (4)	0.1637 (7)	0.1747 (4)	0.029 (2)	
H6BA	0.891536	0.095926	0.176091	0.035*	
C7B	0.8320 (4)	0.2045 (6)	0.1902 (4)	0.0211 (18)	
C8B	0.7685 (5)	0.0613 (6)	0.2132 (5)	0.035 (2)	
H8BA	0.729320	0.050636	0.231820	0.042*	
H8BB	0.797594	0.031982	0.242073	0.042*	
C9B	0.7715 (5)	0.0106 (7)	0.1499 (5)	0.044 (3)	
H9BA	0.804968	0.036029	0.125368	0.052*	
H9BB	0.735353	0.023709	0.125724	0.052*	
C10B	0.7786 (5)	−0.0982 (7)	0.1589 (5)	0.043 (2)	
H10C	0.812544	−0.110195	0.186870	0.051*	
H10D	0.743445	−0.123740	0.180306	0.051*	
C11B	0.7876 (6)	−0.1531 (9)	0.0970 (6)	0.055 (3)	
H11C	0.753890	−0.141305	0.068707	0.066*	
H11D	0.823116	−0.128590	0.075610	0.066*	
C12B	0.7939 (6)	−0.2601 (7)	0.1078 (6)	0.053 (3)	
H12D	0.799873	−0.292617	0.067156	0.079*	
H12E	0.758432	−0.285000	0.127923	0.079*	
H12F	0.827576	−0.272175	0.135354	0.079*	
C13B	0.6811 (3)	0.2286 (5)	0.2425 (4)	0.0191 (16)	
C14B	0.6381 (4)	0.2784 (6)	0.2093 (4)	0.0214 (17)	
H14B	0.648535	0.314712	0.172901	0.026*	
C15B	0.5796 (3)	0.2755 (5)	0.2288 (4)	0.0191 (17)	
C16B	0.5657 (3)	0.2196 (5)	0.2807 (4)	0.0190 (17)	
H16B	0.525957	0.214610	0.293345	0.023*	
C17B	0.6084 (4)	0.1701 (6)	0.3151 (4)	0.0194 (17)	
C18B	0.6664 (4)	0.1748 (6)	0.2958 (4)	0.0183 (16)	
H18B	0.695855	0.141562	0.318779	0.022*	
C19B	0.5324 (4)	0.3299 (6)	0.1905 (5)	0.029 (2)	
C20B	0.5184 (5)	0.2722 (7)	0.1314 (5)	0.037 (2)	
H20G	0.487844	0.305128	0.107264	0.056*	
H20H	0.504703	0.207876	0.143576	0.056*	
H20I	0.553625	0.266173	0.105375	0.056*	
C21B	0.5551 (4)	0.4300 (6)	0.1715 (5)	0.034 (2)	
H21G	0.523637	0.466529	0.151243	0.050*	
H21H	0.587766	0.422880	0.141859	0.050*	
H21I	0.568473	0.464423	0.209314	0.050*	
C22B	0.4769 (4)	0.3428 (7)	0.2295 (6)	0.037 (2)	
H22G	0.448756	0.381674	0.205605	0.056*	
H22H	0.486359	0.375425	0.269295	0.056*	
H22I	0.459799	0.279560	0.238631	0.056*	
C23B	0.5902 (3)	0.1098 (6)	0.3686 (4)	0.0199 (17)	
C24B	0.5475 (4)	0.0013 (6)	0.4250 (4)	0.0238 (18)	
C25B	0.5131 (6)	−0.0741 (9)	0.4486 (6)	0.055 (4)	
H25B	0.487665	−0.109470	0.421671	0.066*	
C26B	0.5173 (7)	−0.0950 (10)	0.5115 (6)	0.063 (4)	
H26B	0.496196	−0.148585	0.527625	0.075*	
C27B	0.5513 (5)	−0.0407 (8)	0.5533 (5)	0.042 (3)	
H27B	0.551430	−0.055549	0.597187	0.050*	
C28B	0.5844 (5)	0.0337 (8)	0.5310 (5)	0.036 (2)	
H28B	0.608254	0.070615	0.558637	0.044*	
C29B	0.5824 (4)	0.0539 (7)	0.4664 (4)	0.0251 (18)	
C30B	0.6566 (7)	0.1804 (14)	0.4558 (8)	0.033 (3)	0.622 (4)
H30C	0.642067	0.212093	0.494658	0.039*	0.622 (4)
H30D	0.667376	0.231478	0.425283	0.039*	0.622 (4)
C31B	0.7112 (6)	0.1213 (13)	0.4720 (8)	0.043 (3)	0.622 (4)
H31C	0.697765	0.064690	0.496501	0.051*	0.622 (4)
H31D	0.726833	0.096338	0.431544	0.051*	0.622 (4)
C32B	0.7619 (7)	0.1635 (13)	0.5075 (9)	0.051 (4)	0.622 (4)
H32C	0.748096	0.185528	0.549417	0.062*	0.622 (4)
H32D	0.776278	0.220603	0.484188	0.062*	0.622 (4)
C33B	0.8125 (8)	0.0937 (15)	0.5169 (11)	0.050 (5)	0.622 (4)
H33C	0.799466	0.038175	0.542684	0.060*	0.622 (4)
H33D	0.825893	0.069148	0.475425	0.060*	0.622 (4)
C34B	0.8615 (10)	0.144 (2)	0.5494 (12)	0.078 (6)	0.622 (4)
H34G	0.893156	0.097697	0.557276	0.117*	0.622 (4)
H34H	0.847678	0.169981	0.589724	0.117*	0.622 (4)
H34I	0.875849	0.196110	0.522596	0.117*	0.622 (4)
C30D	0.6324 (19)	0.225 (4)	0.455 (2)	0.031 (5)	0.224 (4)
H30G	0.651079	0.260090	0.419621	0.037*	0.224 (4)
H30H	0.598334	0.262677	0.469152	0.037*	0.224 (4)
C31D	0.6758 (14)	0.216 (3)	0.5091 (17)	0.036 (4)	0.224 (4)
H31G	0.661647	0.260357	0.542709	0.043*	0.224 (4)
H31H	0.671888	0.150242	0.526046	0.043*	0.224 (4)
C32D	0.7400 (14)	0.235 (3)	0.502 (2)	0.049 (5)	0.224 (4)
H32G	0.746583	0.305185	0.507051	0.059*	0.224 (4)
H32H	0.751535	0.217543	0.458631	0.059*	0.224 (4)
C33D	0.7810 (18)	0.183 (4)	0.548 (2)	0.053 (5)	0.224 (4)
H33G	0.802722	0.230970	0.573246	0.063*	0.224 (4)
H33H	0.757515	0.143207	0.577475	0.063*	0.224 (4)
C34D	0.823 (3)	0.120 (4)	0.514 (3)	0.052 (8)	0.224 (4)
H34J	0.838542	0.071261	0.543323	0.078*	0.224 (4)
H34K	0.855545	0.159038	0.497837	0.078*	0.224 (4)
H34L	0.803466	0.087831	0.478746	0.078*	0.224 (4)
C30E	0.642 (3)	0.213 (5)	0.443 (4)	0.032 (5)	0.154 (3)
H30I	0.660759	0.237768	0.404424	0.038*	0.154 (3)
H30J	0.672984	0.199656	0.474717	0.038*	0.154 (3)
C31E	0.601 (2)	0.290 (3)	0.470 (3)	0.032 (6)	0.154 (3)
H31I	0.576425	0.315794	0.434789	0.038*	0.154 (3)
H31J	0.573973	0.259743	0.500764	0.038*	0.154 (3)
C32E	0.631 (2)	0.374 (3)	0.502 (3)	0.036 (6)	0.154 (3)
H32I	0.663817	0.395621	0.474198	0.043*	0.154 (3)
H32J	0.648870	0.350630	0.541834	0.043*	0.154 (3)
C33E	0.592 (2)	0.461 (4)	0.516 (3)	0.048 (5)	0.154 (3)
H33I	0.574433	0.484567	0.476820	0.057*	0.154 (3)
H33J	0.560275	0.440285	0.545116	0.057*	0.154 (3)
C34E	0.626 (3)	0.540 (4)	0.547 (4)	0.053 (11)	0.154 (3)
H34M	0.664284	0.546349	0.525649	0.079*	0.154 (3)
H34N	0.632801	0.524443	0.591694	0.079*	0.154 (3)
H34O	0.604768	0.600389	0.543507	0.079*	0.154 (3)

### Atomic displacement parameters (Å^2^)

**Table:**

	*U*^11^	*U*^22^	*U*^33^	*U*^12^	*U*^13^	*U*^23^
Hg1	0.02327 (15)	0.01836 (14)	0.0316 (2)	0.00293 (12)	0.00245 (14)	0.00065 (15)
Hg2	0.03127 (17)	0.02021 (14)	0.02125 (17)	−0.00420 (13)	−0.00170 (14)	−0.00320 (15)
Br12	0.0428 (6)	0.0365 (5)	0.0318 (5)	−0.0087 (4)	0.0078 (4)	−0.0070 (4)
Br22	0.0587 (7)	0.0351 (5)	0.0396 (6)	0.0027 (5)	0.0191 (5)	−0.0035 (4)
Br11	0.0125 (18)	0.0248 (11)	0.067 (2)	−0.0023 (11)	0.0074 (14)	0.0025 (10)
Br21	0.0360 (11)	0.019 (3)	0.029 (5)	−0.0054 (18)	−0.008 (4)	0.005 (3)
Cl11	0.0125 (18)	0.0248 (11)	0.067 (2)	−0.0023 (11)	0.0074 (14)	0.0025 (10)
Cl21	0.0360 (11)	0.019 (3)	0.029 (5)	−0.0054 (18)	−0.008 (4)	0.005 (3)
N1A	0.028 (4)	0.032 (4)	0.013 (3)	0.005 (3)	−0.005 (3)	−0.005 (3)
N2A	0.041 (4)	0.031 (4)	0.026 (4)	0.010 (3)	0.002 (3)	0.000 (3)
N3A	0.030 (4)	0.012 (3)	0.022 (4)	−0.005 (3)	−0.004 (3)	−0.001 (3)
N4A	0.050 (5)	0.019 (4)	0.034 (5)	−0.015 (3)	0.012 (4)	−0.007 (3)
C1A	0.030 (4)	0.020 (4)	0.015 (4)	0.000 (3)	0.002 (3)	−0.004 (3)
C2A	0.031 (7)	0.042 (7)	0.022 (6)	0.004 (6)	0.000 (6)	−0.006 (6)
C3A	0.036 (8)	0.049 (8)	0.024 (7)	0.007 (7)	0.002 (7)	−0.007 (7)
C4A	0.041 (8)	0.055 (8)	0.030 (7)	0.010 (8)	0.009 (7)	−0.010 (7)
C5A	0.044 (8)	0.059 (8)	0.026 (7)	0.003 (8)	0.007 (7)	−0.006 (7)
C6A	0.041 (8)	0.055 (8)	0.023 (7)	0.003 (7)	0.005 (7)	−0.002 (7)
C7A	0.037 (7)	0.045 (7)	0.023 (6)	0.004 (6)	0.002 (6)	−0.004 (6)
C2C	0.032 (5)	0.043 (6)	0.022 (5)	0.004 (4)	0.001 (4)	−0.006 (4)
C3C	0.036 (7)	0.047 (7)	0.025 (5)	0.006 (5)	0.005 (5)	−0.008 (5)
C4C	0.042 (7)	0.059 (7)	0.029 (5)	0.008 (7)	0.016 (5)	−0.009 (6)
C5C	0.044 (7)	0.066 (7)	0.022 (5)	0.008 (6)	0.011 (5)	−0.006 (5)
C6C	0.039 (7)	0.060 (7)	0.022 (5)	0.008 (6)	0.006 (5)	−0.004 (5)
C7C	0.035 (6)	0.047 (6)	0.025 (5)	0.006 (5)	0.005 (4)	−0.004 (5)
C8A	0.058 (7)	0.049 (6)	0.023 (5)	0.019 (5)	−0.002 (5)	0.008 (4)
C9A	0.037 (6)	0.097 (10)	0.039 (6)	0.025 (6)	0.012 (5)	0.022 (6)
C10A	0.085 (10)	0.123 (15)	0.059 (9)	0.067 (11)	−0.002 (8)	0.023 (9)
C11A	0.081 (11)	0.17 (2)	0.058 (10)	0.067 (12)	0.007 (8)	0.062 (12)
C12A	0.063 (9)	0.21 (2)	0.033 (8)	0.037 (12)	0.009 (7)	0.049 (11)
C13A	0.040 (5)	0.017 (4)	0.020 (4)	0.004 (4)	0.001 (4)	0.002 (3)
C14A	0.031 (4)	0.017 (3)	0.027 (5)	0.007 (3)	−0.002 (4)	0.000 (4)
C15A	0.042 (5)	0.017 (4)	0.026 (5)	0.003 (4)	−0.006 (4)	0.000 (3)
C16A	0.038 (5)	0.017 (4)	0.022 (5)	0.002 (4)	−0.002 (4)	−0.002 (3)
C17A	0.037 (5)	0.013 (4)	0.017 (4)	−0.004 (3)	0.002 (3)	0.002 (3)
C18A	0.039 (5)	0.015 (4)	0.011 (4)	0.002 (3)	0.002 (3)	−0.003 (3)
C19A	0.039 (5)	0.040 (5)	0.027 (5)	0.010 (4)	−0.015 (4)	−0.008 (4)
C20A	0.042 (10)	0.081 (13)	0.045 (10)	0.012 (10)	−0.024 (8)	−0.021 (10)
C21A	0.040 (9)	0.028 (9)	0.069 (12)	−0.002 (7)	−0.017 (9)	−0.011 (8)
C22A	0.082 (13)	0.075 (13)	0.032 (9)	−0.006 (11)	−0.031 (9)	0.001 (9)
C20C	0.067 (14)	0.058 (14)	0.045 (13)	−0.009 (12)	−0.020 (12)	0.002 (12)
C21C	0.060 (13)	0.063 (14)	0.060 (13)	0.014 (12)	−0.038 (11)	−0.020 (12)
C22C	0.037 (9)	0.058 (10)	0.030 (9)	0.010 (8)	−0.022 (8)	0.004 (8)
C23A	0.037 (5)	0.021 (4)	0.019 (4)	−0.010 (4)	0.006 (4)	−0.006 (3)
C24A	0.039 (5)	0.029 (5)	0.012 (4)	−0.012 (4)	0.004 (4)	−0.001 (3)
C25A	0.035 (5)	0.045 (6)	0.016 (4)	−0.001 (4)	−0.003 (4)	−0.006 (4)
C26A	0.032 (5)	0.054 (6)	0.023 (5)	−0.002 (5)	0.003 (4)	−0.015 (4)
C27A	0.046 (6)	0.059 (7)	0.017 (5)	−0.022 (5)	0.003 (4)	−0.007 (4)
C28A	0.047 (6)	0.037 (6)	0.036 (6)	−0.022 (5)	0.014 (5)	−0.003 (5)
C29A	0.051 (6)	0.029 (5)	0.025 (5)	−0.007 (5)	0.012 (4)	−0.013 (4)
C30A	0.066 (5)	0.024 (4)	0.051 (4)	−0.007 (4)	0.018 (4)	−0.001 (3)
C31A	0.068 (5)	0.028 (4)	0.047 (4)	−0.007 (4)	0.017 (4)	0.006 (4)
C32A	0.067 (5)	0.037 (4)	0.045 (5)	0.001 (4)	0.016 (4)	0.008 (4)
C33A	0.069 (7)	0.047 (7)	0.046 (6)	−0.002 (6)	0.009 (6)	0.006 (6)
C34A	0.068 (10)	0.079 (11)	0.059 (10)	0.007 (9)	0.007 (9)	0.018 (9)
C30C	0.066 (5)	0.024 (4)	0.051 (4)	−0.007 (4)	0.018 (4)	−0.001 (3)
C31C	0.068 (5)	0.028 (4)	0.047 (4)	−0.007 (4)	0.017 (4)	0.006 (4)
C32C	0.067 (5)	0.037 (4)	0.045 (5)	0.001 (4)	0.016 (4)	0.008 (4)
C33C	0.066 (8)	0.044 (8)	0.044 (7)	−0.002 (8)	0.014 (7)	0.008 (7)
C34C	0.061 (14)	0.058 (14)	0.042 (14)	0.002 (14)	0.014 (13)	0.014 (13)
C30F	0.066 (5)	0.024 (4)	0.051 (4)	−0.007 (4)	0.018 (4)	−0.001 (3)
C31F	0.068 (7)	0.031 (6)	0.051 (6)	−0.003 (6)	0.017 (6)	0.002 (6)
C32F	0.068 (7)	0.037 (6)	0.047 (6)	−0.003 (6)	0.014 (6)	0.007 (6)
C33F	0.071 (10)	0.035 (9)	0.047 (9)	−0.006 (9)	0.013 (9)	0.005 (9)
C34F	0.08 (3)	0.05 (3)	0.04 (2)	0.00 (3)	0.00 (2)	−0.01 (2)
N1B	0.026 (4)	0.012 (3)	0.029 (4)	0.009 (3)	0.007 (3)	−0.001 (3)
N2B	0.034 (4)	0.020 (3)	0.013 (3)	0.002 (3)	0.007 (3)	0.001 (3)
N3B	0.023 (3)	0.024 (3)	0.010 (3)	−0.007 (3)	−0.003 (3)	−0.002 (3)
N4B	0.031 (4)	0.028 (4)	0.021 (4)	−0.006 (3)	−0.004 (3)	−0.002 (3)
C1B	0.029 (4)	0.017 (4)	0.015 (4)	0.002 (3)	0.002 (3)	0.001 (3)
C2B	0.023 (4)	0.017 (4)	0.035 (5)	0.003 (3)	0.008 (4)	−0.006 (4)
C3B	0.033 (5)	0.017 (4)	0.052 (6)	−0.002 (4)	0.007 (5)	0.002 (4)
C4B	0.029 (5)	0.036 (5)	0.050 (6)	−0.002 (4)	0.013 (5)	−0.001 (5)
C5B	0.029 (5)	0.038 (5)	0.027 (5)	0.014 (4)	0.009 (4)	0.002 (4)
C6B	0.039 (5)	0.030 (5)	0.020 (4)	0.016 (4)	0.012 (4)	0.006 (4)
C7B	0.029 (5)	0.020 (4)	0.014 (4)	0.003 (4)	0.005 (3)	0.002 (3)
C8B	0.044 (6)	0.017 (4)	0.044 (6)	0.003 (4)	0.021 (4)	−0.003 (4)
C9B	0.056 (7)	0.041 (6)	0.034 (6)	−0.009 (5)	0.011 (5)	−0.009 (5)
C10B	0.049 (6)	0.039 (6)	0.040 (6)	−0.006 (5)	0.007 (5)	−0.001 (5)
C11B	0.054 (7)	0.054 (7)	0.056 (7)	−0.008 (6)	0.011 (6)	−0.014 (6)
C12B	0.081 (9)	0.027 (5)	0.051 (7)	0.022 (6)	−0.021 (6)	−0.023 (5)
C13B	0.023 (4)	0.016 (3)	0.019 (4)	0.000 (3)	0.003 (3)	−0.006 (3)
C14B	0.025 (4)	0.014 (4)	0.025 (5)	−0.001 (3)	0.001 (3)	−0.001 (3)
C15B	0.022 (4)	0.011 (4)	0.024 (4)	−0.001 (3)	−0.010 (3)	−0.006 (3)
C16B	0.015 (3)	0.014 (4)	0.027 (5)	0.002 (3)	0.001 (3)	−0.007 (3)
C17B	0.023 (4)	0.020 (4)	0.016 (4)	−0.001 (3)	−0.003 (3)	−0.009 (3)
C18B	0.025 (4)	0.011 (4)	0.019 (4)	−0.001 (3)	−0.001 (3)	−0.002 (3)
C19B	0.030 (5)	0.020 (4)	0.036 (5)	−0.001 (4)	−0.008 (4)	0.004 (4)
C20B	0.048 (6)	0.029 (5)	0.035 (5)	0.001 (4)	−0.021 (5)	0.001 (4)
C21B	0.037 (5)	0.018 (4)	0.045 (6)	0.001 (4)	−0.006 (4)	0.009 (4)
C22B	0.024 (5)	0.023 (4)	0.065 (7)	0.005 (4)	−0.003 (4)	0.005 (4)
C23B	0.020 (4)	0.021 (4)	0.019 (4)	0.002 (3)	0.003 (3)	−0.001 (3)
C24B	0.033 (5)	0.028 (4)	0.010 (4)	−0.003 (4)	−0.003 (3)	−0.002 (3)
C25B	0.093 (10)	0.045 (7)	0.027 (6)	−0.039 (7)	0.007 (6)	0.002 (5)
C26B	0.098 (11)	0.068 (9)	0.021 (5)	−0.048 (8)	0.005 (6)	0.002 (5)
C27B	0.062 (7)	0.051 (6)	0.012 (4)	−0.008 (5)	−0.007 (4)	0.000 (4)
C28B	0.042 (5)	0.043 (6)	0.024 (5)	−0.006 (5)	−0.012 (4)	−0.004 (4)
C29B	0.025 (4)	0.032 (5)	0.018 (4)	0.003 (4)	0.003 (3)	0.001 (4)
C30B	0.031 (7)	0.046 (8)	0.022 (6)	−0.014 (6)	0.000 (6)	−0.002 (6)
C31B	0.037 (7)	0.059 (8)	0.032 (6)	−0.008 (6)	0.008 (5)	0.019 (6)
C32B	0.066 (8)	0.049 (7)	0.040 (7)	−0.007 (7)	0.017 (6)	0.014 (7)
C33B	0.064 (9)	0.054 (9)	0.032 (7)	−0.004 (8)	0.014 (7)	0.012 (8)
C34B	0.083 (13)	0.085 (13)	0.066 (12)	−0.017 (12)	0.003 (11)	0.013 (11)
C30D	0.034 (10)	0.040 (10)	0.018 (9)	−0.011 (9)	−0.005 (9)	−0.002 (9)
C31D	0.038 (9)	0.047 (9)	0.024 (8)	−0.009 (8)	0.001 (8)	0.006 (8)
C32D	0.054 (9)	0.055 (9)	0.038 (8)	−0.007 (8)	0.009 (8)	0.013 (9)
C33D	0.063 (10)	0.055 (10)	0.041 (9)	−0.004 (9)	0.009 (9)	0.014 (9)
C34D	0.062 (15)	0.059 (16)	0.035 (14)	−0.011 (15)	0.016 (14)	0.016 (14)
C30E	0.034 (10)	0.042 (11)	0.019 (10)	−0.010 (9)	−0.002 (9)	−0.001 (9)
C31E	0.036 (11)	0.040 (11)	0.019 (11)	−0.006 (10)	0.006 (10)	−0.002 (10)
C32E	0.043 (12)	0.040 (12)	0.025 (11)	−0.004 (12)	0.011 (11)	0.006 (11)
C33E	0.058 (10)	0.049 (9)	0.037 (9)	−0.004 (9)	0.011 (9)	0.004 (9)
C34E	0.06 (2)	0.05 (2)	0.04 (2)	0.00 (2)	0.003 (19)	0.008 (19)

### Geometric parameters (Å, º)

**Table:**

Hg1—N1A	2.313 (7)	C34C—H34E	0.9800
Hg1—N1B	2.343 (6)	C34C—H34F	0.9800
Hg1—Cl11	2.373 (19)	C30F—C31F	1.53 (3)
Hg1—Br12	2.5083 (10)	C30F—H30K	0.9900
Hg1—Br11	2.593 (4)	C30F—H30L	0.9900
Hg2—N3A^i^	2.316 (6)	C31F—C32F	1.52 (3)
Hg2—N3B	2.327 (6)	C31F—H31K	0.9900
Hg2—Cl21	2.48 (4)	C31F—H31L	0.9900
Hg2—Br22	2.5147 (11)	C32F—C33F	1.53 (3)
Hg2—Br21	2.57 (2)	C32F—H32K	0.9900
N1A—C1A	1.326 (11)	C32F—H32L	0.9900
N1A—C2A	1.39 (2)	C33F—C34F	1.50 (3)
N1A—C2C	1.408 (15)	C33F—H33K	0.9900
N2A—C1A	1.364 (12)	C33F—H33L	0.9900
N2A—C7A	1.38 (2)	C34F—H34P	0.9800
N2A—C7C	1.384 (15)	C34F—H34Q	0.9800
N2A—C8A	1.460 (13)	C34F—H34R	0.9800
N3A—C23A	1.324 (11)	N1B—C1B	1.340 (11)
N3A—C24A	1.391 (12)	N1B—C2B	1.398 (11)
N4A—C23A	1.372 (12)	N2B—C1B	1.368 (11)
N4A—C29A	1.376 (14)	N2B—C7B	1.388 (11)
N4A—C30F	1.461 (13)	N2B—C8B	1.456 (11)
N4A—C30A	1.461 (13)	N3B—C23B	1.300 (11)
N4A—C30C	1.461 (13)	N3B—C24B	1.396 (12)
C1A—C13A	1.466 (12)	N4B—C23B	1.354 (11)
C2A—C7A	1.38 (3)	N4B—C29B	1.385 (12)
C2A—C3A	1.39 (3)	N4B—C30B	1.465 (17)
C3A—C4A	1.41 (3)	N4B—C30E	1.50 (7)
C3A—H3AA	0.9500	N4B—C30D	1.62 (5)
C4A—C5A	1.36 (3)	C1B—C13B	1.464 (11)
C4A—H4AA	0.9500	C2B—C3B	1.397 (13)
C5A—C6A	1.37 (3)	C2B—C7B	1.400 (12)
C5A—H5AA	0.9500	C3B—C4B	1.375 (14)
C6A—C7A	1.40 (3)	C3B—H3BA	0.9500
C6A—H6AA	0.9500	C4B—C5B	1.407 (14)
C2C—C7C	1.387 (17)	C4B—H4BA	0.9500
C2C—C3C	1.402 (17)	C5B—C6B	1.375 (14)
C3C—C4C	1.40 (2)	C5B—H5BA	0.9500
C3C—H3CA	0.9500	C6B—C7B	1.394 (12)
C4C—C5C	1.36 (2)	C6B—H6BA	0.9500
C4C—H4CA	0.9500	C8B—C9B	1.513 (13)
C5C—C6C	1.374 (19)	C8B—H8BA	0.9900
C5C—H5CA	0.9500	C8B—H8BB	0.9900
C6C—C7C	1.398 (16)	C9B—C10B	1.532 (14)
C6C—H6CA	0.9500	C9B—H9BA	0.9900
C8A—C9A	1.497 (17)	C9B—H9BB	0.9900
C8A—H8AA	0.9900	C10B—C11B	1.528 (14)
C8A—H8AB	0.9900	C10B—H10C	0.9900
C9A—C10A	1.52 (2)	C10B—H10D	0.9900
C9A—H9A1	0.9900	C11B—C12B	1.510 (15)
C9A—H9A2	0.9900	C11B—H11C	0.9900
C10A—C11A	1.53 (2)	C11B—H11D	0.9900
C10A—H10A	0.9900	C12B—H12D	0.9800
C10A—H10B	0.9900	C12B—H12E	0.9800
C11A—C12A	1.49 (3)	C12B—H12F	0.9800
C11A—H11A	0.9900	C13B—C14B	1.392 (12)
C11A—H11B	0.9900	C13B—C18B	1.393 (12)
C12A—H12A	0.9800	C14B—C15B	1.401 (12)
C12A—H12B	0.9800	C14B—H14B	0.9500
C12A—H12C	0.9800	C15B—C16B	1.380 (12)
C13A—C18A	1.391 (13)	C15B—C19B	1.547 (11)
C13A—C14A	1.406 (13)	C16B—C17B	1.401 (12)
C14A—C15A	1.400 (13)	C16B—H16B	0.9500
C14A—H14A	0.9500	C17B—C18B	1.390 (12)
C15A—C16A	1.383 (14)	C17B—C23B	1.467 (12)
C15A—C19A	1.532 (13)	C18B—H18B	0.9500
C16A—C17A	1.383 (13)	C19B—C20B	1.518 (14)
C16A—H16A	0.9500	C19B—C22B	1.526 (14)
C17A—C18A	1.395 (11)	C19B—C21B	1.537 (12)
C17A—C23A	1.466 (13)	C20B—H20G	0.9800
C18A—H18A	0.9500	C20B—H20H	0.9800
C19A—C20C	1.43 (4)	C20B—H20I	0.9800
C19A—C22A	1.47 (3)	C21B—H21G	0.9800
C19A—C21C	1.54 (3)	C21B—H21H	0.9800
C19A—C20A	1.54 (2)	C21B—H21I	0.9800
C19A—C21A	1.55 (2)	C22B—H22G	0.9800
C19A—C22C	1.58 (3)	C22B—H22H	0.9800
C20A—H20A	0.9800	C22B—H22I	0.9800
C20A—H20B	0.9800	C24B—C29B	1.391 (13)
C20A—H20C	0.9800	C24B—C25B	1.402 (14)
C21A—H21A	0.9800	C25B—C26B	1.366 (17)
C21A—H21B	0.9800	C25B—H25B	0.9500
C21A—H21C	0.9800	C26B—C27B	1.398 (16)
C22A—H22A	0.9800	C26B—H26B	0.9500
C22A—H22B	0.9800	C27B—C28B	1.365 (16)
C22A—H22C	0.9800	C27B—H27B	0.9500
C20C—H20D	0.9800	C28B—C29B	1.395 (13)
C20C—H20E	0.9800	C28B—H28B	0.9500
C20C—H20F	0.9800	C30B—C31B	1.534 (19)
C21C—H21D	0.9800	C30B—H30C	0.9900
C21C—H21E	0.9800	C30B—H30D	0.9900
C21C—H21F	0.9800	C31B—C32B	1.500 (19)
C22C—H22D	0.9800	C31B—H31C	0.9900
C22C—H22E	0.9800	C31B—H31D	0.9900
C22C—H22F	0.9800	C32B—C33B	1.52 (2)
C24A—C29A	1.390 (13)	C32B—H32C	0.9900
C24A—C25A	1.406 (14)	C32B—H32D	0.9900
C25A—C26A	1.378 (14)	C33B—C34B	1.48 (2)
C25A—H25A	0.9500	C33B—H33C	0.9900
C26A—C27A	1.385 (16)	C33B—H33D	0.9900
C26A—H26A	0.9500	C34B—H34G	0.9800
C27A—C28A	1.358 (17)	C34B—H34H	0.9800
C27A—H27A	0.9500	C34B—H34I	0.9800
C28A—C29A	1.415 (14)	C30D—C31D	1.52 (2)
C28A—H28A	0.9500	C30D—H30G	0.9900
C30A—C31A	1.559 (15)	C30D—H30H	0.9900
C30A—H30A	0.9900	C31D—C32D	1.50 (2)
C30A—H30B	0.9900	C31D—H31G	0.9900
C31A—C32A	1.519 (17)	C31D—H31H	0.9900
C31A—H31A	0.9900	C32D—C33D	1.53 (3)
C31A—H31B	0.9900	C32D—H32G	0.9900
C32A—C33A	1.544 (18)	C32D—H32H	0.9900
C32A—H32A	0.9900	C33D—C34D	1.49 (3)
C32A—H32B	0.9900	C33D—H33G	0.9900
C33A—C34A	1.51 (2)	C33D—H33H	0.9900
C33A—H33A	0.9900	C34D—H34J	0.9800
C33A—H33B	0.9900	C34D—H34K	0.9800
C34A—H34A	0.9800	C34D—H34L	0.9800
C34A—H34B	0.9800	C30E—C31E	1.53 (3)
C34A—H34C	0.9800	C30E—H30I	0.9900
C30C—C31C	1.559 (15)	C30E—H30J	0.9900
C30C—H30E	0.9900	C31E—C32E	1.52 (3)
C30C—H30F	0.9900	C31E—H31I	0.9900
C31C—C32C	1.519 (17)	C31E—H31J	0.9900
C31C—H31E	0.9900	C32E—C33E	1.53 (3)
C31C—H31F	0.9900	C32E—H32I	0.9900
C32C—C33C	1.53 (3)	C32E—H32J	0.9900
C32C—H32E	0.9900	C33E—C34E	1.50 (3)
C32C—H32F	0.9900	C33E—H33I	0.9900
C33C—C34C	1.50 (3)	C33E—H33J	0.9900
C33C—H33E	0.9900	C34E—H34M	0.9800
C33C—H33F	0.9900	C34E—H34N	0.9800
C34C—H34D	0.9800	C34E—H34O	0.9800
			
N1A—Hg1—N1B	99.4 (3)	H34D—C34C—H34F	109.5
N1A—Hg1—Cl11	107.1 (6)	H34E—C34C—H34F	109.5
N1B—Hg1—Cl11	108.0 (5)	N4A—C30F—C31F	110 (3)
N1A—Hg1—Br12	115.82 (18)	N4A—C30F—H30K	109.6
N1B—Hg1—Br12	111.26 (17)	C31F—C30F—H30K	109.6
Cl11—Hg1—Br12	114.1 (5)	N4A—C30F—H30L	109.6
N1A—Hg1—Br11	103.9 (2)	C31F—C30F—H30L	109.6
N1B—Hg1—Br11	105.2 (2)	H30K—C30F—H30L	108.2
Br12—Hg1—Br11	119.01 (12)	C32F—C31F—C30F	112 (3)
N3A^i^—Hg2—N3B	97.2 (2)	C32F—C31F—H31K	109.3
N3A^i^—Hg2—Cl21	101.7 (8)	C30F—C31F—H31K	109.3
N3B—Hg2—Cl21	105.0 (9)	C32F—C31F—H31L	109.3
N3A^i^—Hg2—Br22	115.13 (18)	C30F—C31F—H31L	109.3
N3B—Hg2—Br22	109.95 (17)	H31K—C31F—H31L	108.0
Cl21—Hg2—Br22	124.1 (7)	C31F—C32F—C33F	110 (3)
N3A^i^—Hg2—Br21	105.3 (5)	C31F—C32F—H32K	109.6
N3B—Hg2—Br21	107.8 (5)	C33F—C32F—H32K	109.6
Br22—Hg2—Br21	119.1 (4)	C31F—C32F—H32L	109.6
C1A—N1A—C2A	106 (2)	C33F—C32F—H32L	109.6
C1A—N1A—C2C	105.2 (10)	H32K—C32F—H32L	108.1
C1A—N1A—Hg1	124.8 (6)	C34F—C33F—C32F	110 (3)
C2A—N1A—Hg1	129 (2)	C34F—C33F—H33K	109.6
C2C—N1A—Hg1	129.4 (9)	C32F—C33F—H33K	109.6
C1A—N2A—C7A	106 (2)	C34F—C33F—H33L	109.6
C1A—N2A—C7C	108.1 (11)	C32F—C33F—H33L	109.6
C1A—N2A—C8A	127.7 (8)	H33K—C33F—H33L	108.2
C7A—N2A—C8A	126 (2)	C33F—C34F—H34P	109.5
C7C—N2A—C8A	123.0 (11)	C33F—C34F—H34Q	109.5
C23A—N3A—C24A	106.5 (7)	H34P—C34F—H34Q	109.5
C23A—N3A—Hg2^ii^	125.9 (6)	C33F—C34F—H34R	109.5
C24A—N3A—Hg2^ii^	125.5 (6)	H34P—C34F—H34R	109.5
C23A—N4A—C29A	107.0 (8)	H34Q—C34F—H34R	109.5
C23A—N4A—C30F	127.6 (9)	C1B—N1B—C2B	106.2 (7)
C29A—N4A—C30F	125.0 (8)	C1B—N1B—Hg1	124.8 (5)
C23A—N4A—C30A	127.6 (9)	C2B—N1B—Hg1	124.7 (5)
C29A—N4A—C30A	125.0 (8)	C1B—N2B—C7B	107.4 (7)
C23A—N4A—C30C	127.6 (9)	C1B—N2B—C8B	128.6 (8)
C29A—N4A—C30C	125.0 (8)	C7B—N2B—C8B	123.9 (7)
N1A—C1A—N2A	111.6 (7)	C23B—N3B—C24B	106.1 (7)
N1A—C1A—C13A	124.8 (8)	C23B—N3B—Hg2	123.6 (5)
N2A—C1A—C13A	123.5 (8)	C24B—N3B—Hg2	129.7 (5)
C7A—C2A—N1A	108 (3)	C23B—N4B—C29B	108.0 (7)
C7A—C2A—C3A	123 (2)	C23B—N4B—C30B	132.5 (9)
N1A—C2A—C3A	129 (4)	C29B—N4B—C30B	118.8 (9)
C2A—C3A—C4A	116 (3)	C23B—N4B—C30E	117 (3)
C2A—C3A—H3AA	122.1	C29B—N4B—C30E	133 (3)
C4A—C3A—H3AA	122.1	C23B—N4B—C30D	122.4 (17)
C5A—C4A—C3A	120 (3)	C29B—N4B—C30D	123.3 (19)
C5A—C4A—H4AA	120.0	N1B—C1B—N2B	111.4 (7)
C3A—C4A—H4AA	120.0	N1B—C1B—C13B	123.1 (7)
C4A—C5A—C6A	125 (3)	N2B—C1B—C13B	125.5 (7)
C4A—C5A—H5AA	117.3	C3B—C2B—N1B	130.5 (8)
C6A—C5A—H5AA	117.3	C3B—C2B—C7B	120.8 (8)
C5A—C6A—C7A	115 (3)	N1B—C2B—C7B	108.8 (7)
C5A—C6A—H6AA	122.5	C4B—C3B—C2B	117.1 (9)
C7A—C6A—H6AA	122.5	C4B—C3B—H3BA	121.4
N2A—C7A—C2A	107 (3)	C2B—C3B—H3BA	121.4
N2A—C7A—C6A	131 (4)	C3B—C4B—C5B	122.2 (9)
C2A—C7A—C6A	121 (2)	C3B—C4B—H4BA	118.9
C7C—C2C—C3C	118.4 (12)	C5B—C4B—H4BA	118.9
C7C—C2C—N1A	109.6 (14)	C6B—C5B—C4B	120.7 (9)
C3C—C2C—N1A	132.0 (15)	C6B—C5B—H5BA	119.7
C4C—C3C—C2C	118.4 (13)	C4B—C5B—H5BA	119.7
C4C—C3C—H3CA	120.8	C5B—C6B—C7B	117.7 (8)
C2C—C3C—H3CA	120.8	C5B—C6B—H6BA	121.1
C5C—C4C—C3C	121.4 (13)	C7B—C6B—H6BA	121.1
C5C—C4C—H4CA	119.3	N2B—C7B—C6B	132.3 (8)
C3C—C4C—H4CA	119.3	N2B—C7B—C2B	106.2 (7)
C4C—C5C—C6C	121.7 (13)	C6B—C7B—C2B	121.4 (8)
C4C—C5C—H5CA	119.2	N2B—C8B—C9B	113.6 (8)
C6C—C5C—H5CA	119.2	N2B—C8B—H8BA	108.8
C5C—C6C—C7C	117.1 (13)	C9B—C8B—H8BA	108.8
C5C—C6C—H6CA	121.5	N2B—C8B—H8BB	108.8
C7C—C6C—H6CA	121.5	C9B—C8B—H8BB	108.8
N2A—C7C—C2C	105.3 (14)	H8BA—C8B—H8BB	107.7
N2A—C7C—C6C	131.7 (16)	C8B—C9B—C10B	110.7 (9)
C2C—C7C—C6C	122.9 (12)	C8B—C9B—H9BA	109.5
N2A—C8A—C9A	113.0 (11)	C10B—C9B—H9BA	109.5
N2A—C8A—H8AA	109.0	C8B—C9B—H9BB	109.5
C9A—C8A—H8AA	109.0	C10B—C9B—H9BB	109.5
N2A—C8A—H8AB	109.0	H9BA—C9B—H9BB	108.1
C9A—C8A—H8AB	109.0	C11B—C10B—C9B	113.6 (9)
H8AA—C8A—H8AB	107.8	C11B—C10B—H10C	108.8
C8A—C9A—C10A	111.0 (14)	C9B—C10B—H10C	108.8
C8A—C9A—H9A1	109.4	C11B—C10B—H10D	108.8
C10A—C9A—H9A1	109.4	C9B—C10B—H10D	108.8
C8A—C9A—H9A2	109.4	H10C—C10B—H10D	107.7
C10A—C9A—H9A2	109.4	C12B—C11B—C10B	112.0 (10)
H9A1—C9A—H9A2	108.0	C12B—C11B—H11C	109.2
C9A—C10A—C11A	114.3 (12)	C10B—C11B—H11C	109.2
C9A—C10A—H10A	108.7	C12B—C11B—H11D	109.2
C11A—C10A—H10A	108.7	C10B—C11B—H11D	109.2
C9A—C10A—H10B	108.7	H11C—C11B—H11D	107.9
C11A—C10A—H10B	108.7	C11B—C12B—H12D	109.5
H10A—C10A—H10B	107.6	C11B—C12B—H12E	109.5
C12A—C11A—C10A	115.5 (18)	H12D—C12B—H12E	109.5
C12A—C11A—H11A	108.4	C11B—C12B—H12F	109.5
C10A—C11A—H11A	108.4	H12D—C12B—H12F	109.5
C12A—C11A—H11B	108.4	H12E—C12B—H12F	109.5
C10A—C11A—H11B	108.4	C14B—C13B—C18B	120.3 (8)
H11A—C11A—H11B	107.5	C14B—C13B—C1B	117.6 (7)
C11A—C12A—H12A	109.5	C18B—C13B—C1B	122.1 (7)
C11A—C12A—H12B	109.5	C13B—C14B—C15B	120.8 (8)
H12A—C12A—H12B	109.5	C13B—C14B—H14B	119.6
C11A—C12A—H12C	109.5	C15B—C14B—H14B	119.6
H12A—C12A—H12C	109.5	C16B—C15B—C14B	118.1 (7)
H12B—C12A—H12C	109.5	C16B—C15B—C19B	121.9 (7)
C18A—C13A—C14A	119.6 (8)	C14B—C15B—C19B	119.9 (8)
C18A—C13A—C1A	121.0 (8)	C15B—C16B—C17B	121.8 (7)
C14A—C13A—C1A	119.4 (8)	C15B—C16B—H16B	119.1
C15A—C14A—C13A	121.0 (8)	C17B—C16B—H16B	119.1
C15A—C14A—H14A	119.5	C18B—C17B—C16B	119.4 (8)
C13A—C14A—H14A	119.5	C18B—C17B—C23B	121.6 (8)
C16A—C15A—C14A	117.6 (8)	C16B—C17B—C23B	118.8 (7)
C16A—C15A—C19A	120.3 (8)	C17B—C18B—C13B	119.5 (8)
C14A—C15A—C19A	122.0 (9)	C17B—C18B—H18B	120.2
C17A—C16A—C15A	122.5 (9)	C13B—C18B—H18B	120.2
C17A—C16A—H16A	118.8	C20B—C19B—C22B	109.3 (8)
C15A—C16A—H16A	118.8	C20B—C19B—C21B	109.5 (8)
C16A—C17A—C18A	119.6 (9)	C22B—C19B—C21B	108.4 (8)
C16A—C17A—C23A	118.6 (8)	C20B—C19B—C15B	108.7 (7)
C18A—C17A—C23A	121.8 (8)	C22B—C19B—C15B	110.9 (8)
C13A—C18A—C17A	119.6 (8)	C21B—C19B—C15B	110.0 (7)
C13A—C18A—H18A	120.2	C19B—C20B—H20G	109.5
C17A—C18A—H18A	120.2	C19B—C20B—H20H	109.5
C20C—C19A—C15A	111.3 (15)	H20G—C20B—H20H	109.5
C22A—C19A—C15A	110.8 (12)	C19B—C20B—H20I	109.5
C20C—C19A—C21C	117 (2)	H20G—C20B—H20I	109.5
C15A—C19A—C21C	109.6 (13)	H20H—C20B—H20I	109.5
C22A—C19A—C20A	108.3 (16)	C19B—C21B—H21G	109.5
C15A—C19A—C20A	114.6 (11)	C19B—C21B—H21H	109.5
C22A—C19A—C21A	107.8 (16)	H21G—C21B—H21H	109.5
C15A—C19A—C21A	109.7 (10)	C19B—C21B—H21I	109.5
C20A—C19A—C21A	105.4 (15)	H21G—C21B—H21I	109.5
C20C—C19A—C22C	109.7 (19)	H21H—C21B—H21I	109.5
C15A—C19A—C22C	105.0 (12)	C19B—C22B—H22G	109.5
C21C—C19A—C22C	103.8 (18)	C19B—C22B—H22H	109.5
C19A—C20A—H20A	109.5	H22G—C22B—H22H	109.5
C19A—C20A—H20B	109.5	C19B—C22B—H22I	109.5
H20A—C20A—H20B	109.5	H22G—C22B—H22I	109.5
C19A—C20A—H20C	109.5	H22H—C22B—H22I	109.5
H20A—C20A—H20C	109.5	N3B—C23B—N4B	112.1 (7)
H20B—C20A—H20C	109.5	N3B—C23B—C17B	124.0 (7)
C19A—C21A—H21A	109.5	N4B—C23B—C17B	124.0 (7)
C19A—C21A—H21B	109.5	C29B—C24B—N3B	109.2 (8)
H21A—C21A—H21B	109.5	C29B—C24B—C25B	119.3 (9)
C19A—C21A—H21C	109.5	N3B—C24B—C25B	131.5 (9)
H21A—C21A—H21C	109.5	C26B—C25B—C24B	117.8 (11)
H21B—C21A—H21C	109.5	C26B—C25B—H25B	121.1
C19A—C22A—H22A	109.5	C24B—C25B—H25B	121.1
C19A—C22A—H22B	109.5	C25B—C26B—C27B	122.7 (11)
H22A—C22A—H22B	109.5	C25B—C26B—H26B	118.6
C19A—C22A—H22C	109.5	C27B—C26B—H26B	118.6
H22A—C22A—H22C	109.5	C28B—C27B—C26B	119.9 (10)
H22B—C22A—H22C	109.5	C28B—C27B—H27B	120.1
C19A—C20C—H20D	109.5	C26B—C27B—H27B	120.1
C19A—C20C—H20E	109.5	C27B—C28B—C29B	118.2 (9)
H20D—C20C—H20E	109.5	C27B—C28B—H28B	120.9
C19A—C20C—H20F	109.5	C29B—C28B—H28B	120.9
H20D—C20C—H20F	109.5	N4B—C29B—C24B	104.6 (8)
H20E—C20C—H20F	109.5	N4B—C29B—C28B	133.3 (9)
C19A—C21C—H21D	109.5	C24B—C29B—C28B	122.0 (9)
C19A—C21C—H21E	109.5	N4B—C30B—C31B	113.0 (14)
H21D—C21C—H21E	109.5	N4B—C30B—H30C	109.0
C19A—C21C—H21F	109.5	C31B—C30B—H30C	109.0
H21D—C21C—H21F	109.5	N4B—C30B—H30D	109.0
H21E—C21C—H21F	109.5	C31B—C30B—H30D	109.0
C19A—C22C—H22D	109.5	H30C—C30B—H30D	107.8
C19A—C22C—H22E	109.5	C32B—C31B—C30B	122.3 (15)
H22D—C22C—H22E	109.5	C32B—C31B—H31C	106.8
C19A—C22C—H22F	109.5	C30B—C31B—H31C	106.8
H22D—C22C—H22F	109.5	C32B—C31B—H31D	106.8
H22E—C22C—H22F	109.5	C30B—C31B—H31D	106.8
N3A—C23A—N4A	111.3 (8)	H31C—C31B—H31D	106.6
N3A—C23A—C17A	123.7 (8)	C31B—C32B—C33B	114.0 (15)
N4A—C23A—C17A	124.9 (8)	C31B—C32B—H32C	108.8
C29A—C24A—N3A	108.5 (9)	C33B—C32B—H32C	108.8
C29A—C24A—C25A	120.0 (9)	C31B—C32B—H32D	108.8
N3A—C24A—C25A	131.4 (8)	C33B—C32B—H32D	108.8
C26A—C25A—C24A	116.9 (10)	H32C—C32B—H32D	107.7
C26A—C25A—H25A	121.5	C34B—C33B—C32B	109.8 (18)
C24A—C25A—H25A	121.5	C34B—C33B—H33C	109.7
C25A—C26A—C27A	121.8 (10)	C32B—C33B—H33C	109.7
C25A—C26A—H26A	119.1	C34B—C33B—H33D	109.7
C27A—C26A—H26A	119.1	C32B—C33B—H33D	109.7
C28A—C27A—C26A	123.3 (10)	H33C—C33B—H33D	108.2
C28A—C27A—H27A	118.4	C33B—C34B—H34G	109.5
C26A—C27A—H27A	118.4	C33B—C34B—H34H	109.5
C27A—C28A—C29A	115.3 (9)	H34G—C34B—H34H	109.5
C27A—C28A—H28A	122.4	C33B—C34B—H34I	109.5
C29A—C28A—H28A	122.4	H34G—C34B—H34I	109.5
N4A—C29A—C24A	106.6 (9)	H34H—C34B—H34I	109.5
N4A—C29A—C28A	130.7 (9)	C31D—C30D—N4B	114 (4)
C24A—C29A—C28A	122.7 (10)	C31D—C30D—H30G	108.8
N4A—C30A—C31A	112.8 (9)	N4B—C30D—H30G	108.8
N4A—C30A—H30A	109.0	C31D—C30D—H30H	108.8
C31A—C30A—H30A	109.0	N4B—C30D—H30H	108.8
N4A—C30A—H30B	109.0	H30G—C30D—H30H	107.7
C31A—C30A—H30B	109.0	C32D—C31D—C30D	124 (3)
H30A—C30A—H30B	107.8	C32D—C31D—H31G	106.4
C32A—C31A—C30A	115.4 (10)	C30D—C31D—H31G	106.4
C32A—C31A—H31A	108.4	C32D—C31D—H31H	106.4
C30A—C31A—H31A	108.4	C30D—C31D—H31H	106.4
C32A—C31A—H31B	108.4	H31G—C31D—H31H	106.5
C30A—C31A—H31B	108.4	C31D—C32D—C33D	117 (3)
H31A—C31A—H31B	107.5	C31D—C32D—H32G	108.0
C31A—C32A—C33A	111.6 (11)	C33D—C32D—H32G	108.0
C31A—C32A—H32A	109.3	C31D—C32D—H32H	108.0
C33A—C32A—H32A	109.3	C33D—C32D—H32H	108.0
C31A—C32A—H32B	109.3	H32G—C32D—H32H	107.2
C33A—C32A—H32B	109.3	C34D—C33D—C32D	112 (3)
H32A—C32A—H32B	108.0	C34D—C33D—H33G	109.2
C34A—C33A—C32A	109.9 (14)	C32D—C33D—H33G	109.2
C34A—C33A—H33A	109.7	C34D—C33D—H33H	109.2
C32A—C33A—H33A	109.7	C32D—C33D—H33H	109.2
C34A—C33A—H33B	109.7	H33G—C33D—H33H	107.9
C32A—C33A—H33B	109.7	C33D—C34D—H34J	109.5
H33A—C33A—H33B	108.2	C33D—C34D—H34K	109.5
C33A—C34A—H34A	109.5	H34J—C34D—H34K	109.5
C33A—C34A—H34B	109.5	C33D—C34D—H34L	109.5
H34A—C34A—H34B	109.5	H34J—C34D—H34L	109.5
C33A—C34A—H34C	109.5	H34K—C34D—H34L	109.5
H34A—C34A—H34C	109.5	N4B—C30E—C31E	111 (5)
H34B—C34A—H34C	109.5	N4B—C30E—H30I	109.4
N4A—C30C—C31C	112.8 (9)	C31E—C30E—H30I	109.4
N4A—C30C—H30E	109.0	N4B—C30E—H30J	109.4
C31C—C30C—H30E	109.0	C31E—C30E—H30J	109.4
N4A—C30C—H30F	109.0	H30I—C30E—H30J	108.0
C31C—C30C—H30F	109.0	C32E—C31E—C30E	114 (3)
H30E—C30C—H30F	107.8	C32E—C31E—H31I	108.7
C32C—C31C—C30C	115.4 (10)	C30E—C31E—H31I	108.7
C32C—C31C—H31E	108.4	C32E—C31E—H31J	108.7
C30C—C31C—H31E	108.4	C30E—C31E—H31J	108.7
C32C—C31C—H31F	108.4	H31I—C31E—H31J	107.6
C30C—C31C—H31F	108.4	C31E—C32E—C33E	115 (3)
H31E—C31C—H31F	107.5	C31E—C32E—H32I	108.6
C31C—C32C—C33C	128 (3)	C33E—C32E—H32I	108.6
C31C—C32C—H32E	105.4	C31E—C32E—H32J	108.6
C33C—C32C—H32E	105.4	C33E—C32E—H32J	108.6
C31C—C32C—H32F	105.4	H32I—C32E—H32J	107.6
C33C—C32C—H32F	105.4	C34E—C33E—C32E	111 (3)
H32E—C32C—H32F	106.0	C34E—C33E—H33I	109.5
C34C—C33C—C32C	109 (3)	C32E—C33E—H33I	109.5
C34C—C33C—H33E	109.9	C34E—C33E—H33J	109.5
C32C—C33C—H33E	109.9	C32E—C33E—H33J	109.5
C34C—C33C—H33F	109.9	H33I—C33E—H33J	108.0
C32C—C33C—H33F	109.9	C33E—C34E—H34M	109.5
H33E—C33C—H33F	108.3	C33E—C34E—H34N	109.5
C33C—C34C—H34D	109.5	H34M—C34E—H34N	109.5
C33C—C34C—H34E	109.5	C33E—C34E—H34O	109.5
H34D—C34C—H34E	109.5	H34M—C34E—H34O	109.5
C33C—C34C—H34F	109.5	H34N—C34E—H34O	109.5
			
C2A—N1A—C1A—N2A	−5.7 (12)	C23A—N4A—C30A—C31A	102.0 (12)
C2C—N1A—C1A—N2A	4.0 (10)	C29A—N4A—C30A—C31A	−70.7 (14)
Hg1—N1A—C1A—N2A	176.1 (6)	N4A—C30A—C31A—C32A	−61.3 (16)
C2A—N1A—C1A—C13A	172.5 (11)	C30A—C31A—C32A—C33A	−172.0 (14)
C2C—N1A—C1A—C13A	−177.8 (9)	C31A—C32A—C33A—C34A	173.1 (16)
Hg1—N1A—C1A—C13A	−5.8 (12)	C23A—N4A—C30C—C31C	102.0 (12)
C7A—N2A—C1A—N1A	6 (2)	C29A—N4A—C30C—C31C	−70.7 (14)
C7C—N2A—C1A—N1A	−3.1 (13)	N4A—C30C—C31C—C32C	−61.3 (16)
C8A—N2A—C1A—N1A	−170.6 (9)	C30C—C31C—C32C—C33C	−176 (3)
C7A—N2A—C1A—C13A	−173 (2)	C31C—C32C—C33C—C34C	61 (8)
C7C—N2A—C1A—C13A	178.7 (11)	C23A—N4A—C30F—C31F	71 (2)
C8A—N2A—C1A—C13A	11.2 (15)	C29A—N4A—C30F—C31F	−102 (2)
C1A—N1A—C2A—C7A	3 (2)	N4A—C30F—C31F—C32F	48 (5)
Hg1—N1A—C2A—C7A	−178 (2)	C30F—C31F—C32F—C33F	68 (7)
C1A—N1A—C2A—C3A	−176 (2)	C31F—C32F—C33F—C34F	−172 (6)
Hg1—N1A—C2A—C3A	2 (2)	C2B—N1B—C1B—N2B	0.0 (10)
C7A—C2A—C3A—C4A	0 (4)	Hg1—N1B—C1B—N2B	−157.5 (6)
N1A—C2A—C3A—C4A	180 (2)	C2B—N1B—C1B—C13B	−179.8 (8)
C2A—C3A—C4A—C5A	1 (5)	Hg1—N1B—C1B—C13B	22.7 (12)
C3A—C4A—C5A—C6A	−2 (6)	C7B—N2B—C1B—N1B	0.5 (10)
C4A—C5A—C6A—C7A	2 (6)	C8B—N2B—C1B—N1B	−177.0 (9)
C1A—N2A—C7A—C2A	−3 (3)	C7B—N2B—C1B—C13B	−179.7 (8)
C8A—N2A—C7A—C2A	173.2 (15)	C8B—N2B—C1B—C13B	2.8 (14)
C1A—N2A—C7A—C6A	177 (4)	C1B—N1B—C2B—C3B	179.9 (11)
C8A—N2A—C7A—C6A	−7 (6)	Hg1—N1B—C2B—C3B	−22.6 (15)
N1A—C2A—C7A—N2A	0 (3)	C1B—N1B—C2B—C7B	−0.6 (10)
C3A—C2A—C7A—N2A	180 (2)	Hg1—N1B—C2B—C7B	157.0 (6)
N1A—C2A—C7A—C6A	180 (3)	N1B—C2B—C3B—C4B	178.0 (10)
C3A—C2A—C7A—C6A	0 (5)	C7B—C2B—C3B—C4B	−1.6 (16)
C5A—C6A—C7A—N2A	179 (4)	C2B—C3B—C4B—C5B	0.9 (17)
C5A—C6A—C7A—C2A	−1 (5)	C3B—C4B—C5B—C6B	−0.5 (17)
C1A—N1A—C2C—C7C	−3.5 (11)	C4B—C5B—C6B—C7B	0.9 (15)
Hg1—N1A—C2C—C7C	−175.1 (9)	C1B—N2B—C7B—C6B	177.4 (9)
C1A—N1A—C2C—C3C	174.8 (12)	C8B—N2B—C7B—C6B	−4.9 (15)
Hg1—N1A—C2C—C3C	3.3 (14)	C1B—N2B—C7B—C2B	−0.8 (10)
C7C—C2C—C3C—C4C	−1 (2)	C8B—N2B—C7B—C2B	176.8 (8)
N1A—C2C—C3C—C4C	−179.3 (12)	C5B—C6B—C7B—N2B	−179.6 (9)
C2C—C3C—C4C—C5C	0 (3)	C5B—C6B—C7B—C2B	−1.6 (14)
C3C—C4C—C5C—C6C	3 (3)	C3B—C2B—C7B—N2B	−179.5 (9)
C4C—C5C—C6C—C7C	−4 (3)	N1B—C2B—C7B—N2B	0.9 (10)
C1A—N2A—C7C—C2C	0.7 (15)	C3B—C2B—C7B—C6B	2.0 (15)
C8A—N2A—C7C—C2C	169.0 (10)	N1B—C2B—C7B—C6B	−177.6 (8)
C1A—N2A—C7C—C6C	−175.3 (18)	C1B—N2B—C8B—C9B	113.5 (10)
C8A—N2A—C7C—C6C	−7 (3)	C7B—N2B—C8B—C9B	−63.7 (13)
C3C—C2C—C7C—N2A	−176.9 (11)	N2B—C8B—C9B—C10B	162.5 (9)
N1A—C2C—C7C—N2A	1.7 (14)	C8B—C9B—C10B—C11B	−174.7 (10)
C3C—C2C—C7C—C6C	0 (2)	C9B—C10B—C11B—C12B	−179.5 (11)
N1A—C2C—C7C—C6C	178.1 (14)	N1B—C1B—C13B—C14B	49.8 (12)
C5C—C6C—C7C—N2A	178.3 (17)	N2B—C1B—C13B—C14B	−129.9 (9)
C5C—C6C—C7C—C2C	3 (3)	N1B—C1B—C13B—C18B	−127.5 (9)
C1A—N2A—C8A—C9A	−115.5 (11)	N2B—C1B—C13B—C18B	52.7 (12)
C7A—N2A—C8A—C9A	69 (3)	C18B—C13B—C14B—C15B	0.2 (12)
C7C—N2A—C8A—C9A	78.6 (15)	C1B—C13B—C14B—C15B	−177.2 (7)
N2A—C8A—C9A—C10A	−166.8 (10)	C13B—C14B—C15B—C16B	−2.0 (11)
C8A—C9A—C10A—C11A	76.3 (19)	C13B—C14B—C15B—C19B	−178.9 (7)
C9A—C10A—C11A—C12A	67.4 (19)	C14B—C15B—C16B—C17B	2.8 (11)
N1A—C1A—C13A—C18A	−119.2 (10)	C19B—C15B—C16B—C17B	179.7 (7)
N2A—C1A—C13A—C18A	58.7 (12)	C15B—C16B—C17B—C18B	−1.8 (12)
N1A—C1A—C13A—C14A	62.0 (12)	C15B—C16B—C17B—C23B	−178.3 (7)
N2A—C1A—C13A—C14A	−120.1 (10)	C16B—C17B—C18B—C13B	0.0 (11)
C18A—C13A—C14A—C15A	−1.2 (12)	C23B—C17B—C18B—C13B	176.3 (7)
C1A—C13A—C14A—C15A	177.6 (8)	C14B—C13B—C18B—C17B	0.8 (11)
C13A—C14A—C15A—C16A	1.0 (13)	C1B—C13B—C18B—C17B	178.1 (7)
C13A—C14A—C15A—C19A	−179.8 (8)	C16B—C15B—C19B—C20B	−99.9 (10)
C14A—C15A—C16A—C17A	0.6 (13)	C14B—C15B—C19B—C20B	77.0 (10)
C19A—C15A—C16A—C17A	−178.6 (8)	C16B—C15B—C19B—C22B	20.3 (11)
C15A—C16A—C17A—C18A	−2.2 (13)	C14B—C15B—C19B—C22B	−162.9 (8)
C15A—C16A—C17A—C23A	179.8 (8)	C16B—C15B—C19B—C21B	140.2 (8)
C14A—C13A—C18A—C17A	−0.3 (12)	C14B—C15B—C19B—C21B	−43.0 (11)
C1A—C13A—C18A—C17A	−179.1 (7)	C24B—N3B—C23B—N4B	0.4 (10)
C16A—C17A—C18A—C13A	2.0 (12)	Hg2—N3B—C23B—N4B	−171.3 (5)
C23A—C17A—C18A—C13A	180.0 (8)	C24B—N3B—C23B—C17B	−179.3 (8)
C16A—C15A—C19A—C20C	−139.5 (19)	Hg2—N3B—C23B—C17B	9.0 (11)
C14A—C15A—C19A—C20C	41 (2)	C29B—N4B—C23B—N3B	−0.9 (10)
C16A—C15A—C19A—C22A	61.1 (17)	C30B—N4B—C23B—N3B	169.3 (12)
C14A—C15A—C19A—C22A	−118.1 (16)	C30E—N4B—C23B—N3B	−167 (3)
C16A—C15A—C19A—C21C	−9 (2)	C30D—N4B—C23B—N3B	−154 (2)
C14A—C15A—C19A—C21C	171.7 (17)	C29B—N4B—C23B—C17B	178.8 (8)
C16A—C15A—C19A—C20A	−176.0 (15)	C30B—N4B—C23B—C17B	−11.0 (17)
C14A—C15A—C19A—C20A	4.8 (18)	C30E—N4B—C23B—C17B	12 (3)
C16A—C15A—C19A—C21A	−57.8 (15)	C30D—N4B—C23B—C17B	26 (2)
C14A—C15A—C19A—C21A	123.1 (13)	C18B—C17B—C23B—N3B	−118.7 (9)
C16A—C15A—C19A—C22C	101.9 (15)	C16B—C17B—C23B—N3B	57.6 (11)
C14A—C15A—C19A—C22C	−77.3 (15)	C18B—C17B—C23B—N4B	61.6 (11)
C24A—N3A—C23A—N4A	0.8 (10)	C16B—C17B—C23B—N4B	−122.0 (9)
Hg2^ii^—N3A—C23A—N4A	−163.3 (6)	C23B—N3B—C24B—C29B	0.3 (10)
C24A—N3A—C23A—C17A	−175.7 (8)	Hg2—N3B—C24B—C29B	171.3 (6)
Hg2^ii^—N3A—C23A—C17A	20.2 (12)	C23B—N3B—C24B—C25B	178.7 (12)
C29A—N4A—C23A—N3A	−0.8 (11)	Hg2—N3B—C24B—C25B	−10.3 (16)
C30F—N4A—C23A—N3A	−174.5 (9)	C29B—C24B—C25B—C26B	−2.6 (19)
C30A—N4A—C23A—N3A	−174.5 (9)	N3B—C24B—C25B—C26B	179.2 (12)
C30C—N4A—C23A—N3A	−174.5 (9)	C24B—C25B—C26B—C27B	4 (2)
C29A—N4A—C23A—C17A	175.6 (9)	C25B—C26B—C27B—C28B	−3 (2)
C30F—N4A—C23A—C17A	1.9 (15)	C26B—C27B—C28B—C29B	0.9 (18)
C30A—N4A—C23A—C17A	1.9 (15)	C23B—N4B—C29B—C24B	1.1 (10)
C30C—N4A—C23A—C17A	1.9 (15)	C30B—N4B—C29B—C24B	−170.7 (11)
C16A—C17A—C23A—N3A	57.1 (12)	C30E—N4B—C29B—C24B	165 (3)
C18A—C17A—C23A—N3A	−120.9 (9)	C30D—N4B—C29B—C24B	154 (2)
C16A—C17A—C23A—N4A	−118.9 (10)	C23B—N4B—C29B—C28B	−178.6 (10)
C18A—C17A—C23A—N4A	63.1 (12)	C30B—N4B—C29B—C28B	9.6 (17)
C23A—N3A—C24A—C29A	−0.5 (10)	C30E—N4B—C29B—C28B	−15 (4)
Hg2^ii^—N3A—C24A—C29A	163.7 (6)	C30D—N4B—C29B—C28B	−26 (2)
C23A—N3A—C24A—C25A	179.1 (9)	N3B—C24B—C29B—N4B	−0.9 (10)
Hg2^ii^—N3A—C24A—C25A	−16.7 (13)	C25B—C24B—C29B—N4B	−179.4 (10)
C29A—C24A—C25A—C26A	−1.2 (13)	N3B—C24B—C29B—C28B	178.9 (9)
N3A—C24A—C25A—C26A	179.2 (9)	C25B—C24B—C29B—C28B	0.3 (15)
C24A—C25A—C26A—C27A	−0.6 (14)	C27B—C28B—C29B—N4B	−179.8 (11)
C25A—C26A—C27A—C28A	1.7 (16)	C27B—C28B—C29B—C24B	0.5 (16)
C26A—C27A—C28A—C29A	−0.8 (16)	C23B—N4B—C30B—C31B	−102.4 (15)
C23A—N4A—C29A—C24A	0.4 (11)	C29B—N4B—C30B—C31B	67.0 (15)
C30F—N4A—C29A—C24A	174.4 (9)	N4B—C30B—C31B—C32B	−172.4 (13)
C30A—N4A—C29A—C24A	174.4 (9)	C30B—C31B—C32B—C33B	−178.2 (16)
C30C—N4A—C29A—C24A	174.4 (9)	C31B—C32B—C33B—C34B	177.1 (17)
C23A—N4A—C29A—C28A	178.5 (11)	C23B—N4B—C30D—C31D	−154 (2)
C30F—N4A—C29A—C28A	−7.6 (18)	C29B—N4B—C30D—C31D	57 (4)
C30A—N4A—C29A—C28A	−7.6 (18)	N4B—C30D—C31D—C32D	106 (5)
C30C—N4A—C29A—C28A	−7.6 (18)	C30D—C31D—C32D—C33D	−153 (5)
N3A—C24A—C29A—N4A	0.0 (11)	C31D—C32D—C33D—C34D	120 (5)
C25A—C24A—C29A—N4A	−179.6 (8)	C23B—N4B—C30E—C31E	94 (5)
N3A—C24A—C29A—C28A	−178.2 (9)	C29B—N4B—C30E—C31E	−68 (7)
C25A—C24A—C29A—C28A	2.1 (15)	N4B—C30E—C31E—C32E	165 (5)
C27A—C28A—C29A—N4A	−178.9 (10)	C30E—C31E—C32E—C33E	168 (6)
C27A—C28A—C29A—C24A	−1.1 (16)	C31E—C32E—C33E—C34E	−179 (6)

Symmetry codes: (i) *x*, *y*−1, *z*; (ii) *x*, *y*+1, *z*.

### Hydrogen-bond geometry (Å, º)

**Table:**

*D*—H···*A*	*D*—H	H···*A*	*D*···*A*	*D*—H···*A*
C3*A*—H3*AA*···N1*B*	0.95	2.45	3.29 (4)	148
C8*A*—H8*AB*···Br22^ii^	0.99	2.94	3.873 (11)	158
C25*A*—H25*A*···Br21^ii^	0.95	2.96	3.66 (2)	131
C25*A*—H25*A*···Cl21^ii^	0.95	2.74	3.45 (3)	132
C28*A*—H28*A*···Br11^iii^	0.95	2.96	3.793 (12)	147
C28*A*—H28*A*···Cl11^iii^	0.95	2.86	3.70 (2)	149
C30*A*—H30*B*···Br11^iii^	0.99	3.04	3.979 (13)	158
C3*B*—H3*BA*···Br11	0.95	2.96	3.631 (11)	129
C6*B*—H6*BA*···Br21^iv^	0.95	2.88	3.75 (2)	153
C6*B*—H6*BA*···Cl21^iv^	0.95	2.89	3.73 (4)	149
C25*B*—H25*B*···N3*A*^i^	0.95	2.56	3.332 (14)	139
C30*D*—H30*G*···Br12	0.99	3.06	3.95 (5)	150

Symmetry codes: (i) *x*, *y*−1, *z*; (ii) *x*, *y*+1, *z*; (iii) *x*−1/2, −*y*+1, *z*; (iv) *x*+1/2, −*y*, *z*.

## References

[bb1] Bouchouit, M., Benzerka, S., Bouraiou, A., Merazig, H., Belfaitah, A. & Bouacida, S. (2015). *Acta Cryst.* E**71**, m253–m254. 10.1107/S205698901502349X PMC4719860 26870451

[bb2] Bruker (2002). *SAINT*. Bruker AXS Inc., Madison, Wisconsin, USA.

[bb3] Bruker (2005). *APEX2*. Bruker AXS Inc., Madison, Wisconsin, USA.

[bb4] Carballo, R., Castineiras, A., Conde, M. C. G. & Hiller, W. (1993). *Polyhedron*, **12**, 1655–1660.

[bb5] Carina, R. F., Williams, A. F. & Bernardinelli, G. (1997). *J. Organomet. Chem.* **548**, 45–48.

[bb6] Chen, Y., Chen, C., Chen, H., Cao, T., Yue, Z., Liu, X. & Niu, Y. (2013). *Synth. React. Inorg. Met.-Org. Nano-Met. Chem.* **43**, 1307–1310.

[bb7] Chen, S., Fan, R.-Q., Wang, X.-M. & Yang, Y.-L. (2014). *CrystEngComm*, **16**, 6114–6125.

[bb8] Dey, A., Mandal, S. K. & Biradha, K. (2013). *CrystEngComm*, **15**, 9769–9778.

[bb9] Ding, Y., Zhou, X., Jin, G., Zhao, D. & Meng, X. (2012). *Synth. React. Inorg. Met.-Org. Nano-Met. Chem.* **42**, 438–443.

[bb10] Du, J.-L., Wei, Z.-Z. & Hu, T.-L. (2011). *Solid State Sci.* **13**, 1256–1260.

[bb11] Gonzalez, A. D. (2014). *Organometallics*, **33**, 868–875.

[bb12] He, C.-J., Zu, E.-P. & Zhou, X.-J. (2012). *Z. Kristallogr. New Cryst. Struct.* **227**, 445–446.

[bb13] Hu, J. Y., Liao, C. L., Hu, L. L., Zhang, C. C., Chen, S. F. & Zhao, J. (2015). *Russ. J. Coord. Chem.* **41**, 212–219.

[bb14] Hu, J., Liao, C., Zhao, J. & Haipeng Zhao, H. (2012). *Z. Kristallogr. New Cryst. Struct.* **227**, 69–70.

[bb15] Huang, M., Liu, P., Chen, Y., Wang, J. & Liu, Z. (2006). *J. Mol. Struct.* **788**, 211–217.

[bb16] Karlsson, E. A., Lee, B., Åkermark, T., Johnston, E. V., Kärkäs, M. D., Sun, J., Hansson, Ö., Bäckvall, J. & Åkermark, B. (2011). *Angew. Chem. Int. Ed.* **50**, 11715–11718. 10.1002/anie.201104355 21983946

[bb17] Li, J., Li, X., Lu, H., Zhu, Y., Sun, H., Guo, Y., Yue, Z., Zhao, J., Tang, M., Hou, H., Fan, Y. & Chang, J. (2012*b*). *Inorg. Chim. Acta*, **384**, 163–169.

[bb18] Li, Y., Liu, Q.-K., Ma, J.-P. & Dong, Y.-B. (2012*a*). *Acta Cryst.* C**68**, m152–m155. 10.1107/S0108270112019233 22669186

[bb19] Li, X.-P., Zhang, J.-Y., Liu, Y., Pan, M., Zheng, S.-R., Kang, B.-S. & Cheng-Yong Su, C.-Y. (2007). *Inorg. Chim. Acta*, **360**, 2990–2996.

[bb20] Lou, S.-F., Wang, Q. & Ding, J. (2012). *Z. Kristallogr. New Cryst. Struct.* **227**, 105–106.

[bb21] Manjunatha, M. N., Dikundwar, A. G. & Nagasundara, K. R. (2011). *Polyhedron*, **30**, 1299–1304.

[bb22] Matthews, C. J., Clegg, W., Heath, S. L., Martin, N. C., Hill, M. N. S. & Lockhart, J. C. (1998). *Inorg. Chem.* **37**, 199–207.

[bb23] Obara, S., Itabashi, M., Okuda, F., Tamaki, S., Tanabe, Y., Ishii, Y., Nozaki, K. & Haga, M. (2006). *Inorg. Chem.* **45**, 8907–8921. 10.1021/ic060796o 17054350

[bb24] Palatinus, L. & Chapuis, G. (2007). *J. Appl. Cryst.* **40**, 786–790.

[bb25] Quiroz-Castro, E., Bernes, S., Barba-Behrens, N., Tapia-Benavides, R., Contreras, R. & Noth, H. (2000). *Polyhedron*, **19**, 1479–1484.

[bb26] Rani, V., Singh, H. B. & Butcher, R. J. (2017). *Acta Cryst.* E**73**, 341–344. 10.1107/S2056989017001888 PMC5347049 28316804

[bb27] Rüttimann, S., Bernardinelli, G. & Williams, A. F. (1993). *Angew. Chem. Int. Ed.* **32**, 392–394.

[bb28] Rüttimann, S., Piguet, C., Bernardinelli, G., Bocquet, B. & Williams, A. F. (1992). *J. Am. Chem. Soc.* **114**, 4230–4237.

[bb29] Sheldrick, G. M. (1996). *SADABS*. University of Göttingen, Germany.

[bb30] Sheldrick, G. M. (2008). *Acta Cryst.* A**64**, 112–122. 10.1107/S0108767307043930 18156677

[bb31] Sheldrick, G. M. (2015). *Acta Cryst.* C**71**, 3–8.

[bb32] Shen, Y.-H., Liu, J.-G. & Xu, D.-J. (2005). *Acta Cryst.* E**61**, m1880–m1882.

[bb33] Su, C.-Y., Goforth, A. M., Smith, M. D. & zur Loye, H.-C. (2003). *Inorg. Chem.* **42**, 5685–5692. 10.1021/ic034388l 12950218

[bb34] Tam, A. Y. Y., Tsang, D. P. K., Chan, M. Y., Zhu, N. & Yam, V. W. W. (2012). *Chem. Commun.* **47**, 3383–3385. 10.1039/c0cc05538g 21327274

[bb35] Wang, Q., Fu, Z.-Y. & Yu, L.-M. (2012). *Acta Cryst.* E**68**, m44. 10.1107/S1600536811051166 PMC3254313 22259344

[bb36] Wang, X.-F., Lv, Y., Su, Z., Okamura, T., Wu, G., Sun, W.-Y. & Ueyama, N. (2007). *Z. Anorg. Allg. Chem.* **633**, 2695–2700.

[bb37] Wang, J.-J., Yan, L.-F., Li, Z.-X., Chang, Z., Hu, T.-L. & Bu, X.-H. (2009). *Inorg. Chim. Acta*, **362**, 3147–3154.

[bb38] Wang, X., Yang, H.-Y., Zhang, C., Yuan, J. & Yang, H.-X. (2015). *Z. Kristallogr. New Cryst. Struct.* **230**, 361–362.

[bb39] Wu, J., Yang, J. & Pan, F. (2009). *Acta Cryst.* E**65**, m829. 10.1107/S1600536809023459 PMC2969412 21582747

[bb40] Xiao, B., Li, W., Hou, H. & Fan, Y. (2009). *J. Coord. Chem.* **62**, 1630–1637.

[bb41] Xiao, B., Yang, L.-J., Xiao, H.-Y. & Fang, S.-M. (2011). *J. Coord. Chem.* **64**, 4408–4420.

[bb42] Xie, Q., Liu, S., Li, X., Wu, Q., Luo, Z., Fu, X., Cao, W., Lan, G., Li, D., Zheng, W. & Chen, T. (2014). *Dalton Trans.* **43**, 6973–6976. 10.1039/c4dt00198b 24668337

[bb43] Xu, C., Wang, X., Ding, D., Hou, H. & Fan, Y. (2011). *Inorg. Chem. Commun.* **14**, 1410–1413.

[bb44] Yan, S., Jin, G., Yang, Y., Su, X. & Meng, X. (2012). *Synth. React. Inorg. Met.-Org. Nano-Met. Chem.* **42**, 678–684.

[bb45] Yang, G.-Y. & Luo, L.-X. (2012). *Z. Kristallogr. New Cryst. Struct.* **227**, 441–442.

[bb46] Yang, W. W., Zhong, Y. W., Yoshikawa, S., Shao, J. Y., Masaoka, S., Sakai, K., Yao, J. & Haga, M. (2012). *Inorg. Chem.* **51**, 890–899. 10.1021/ic2016885 22206350

[bb47] Zhang, Z., Feng, Y.-F., Wei, Q.-Y., Hu, K., Chen, Z.-L. & Liang, F.-P. (2015). *CrystEngComm*, **17**, 6724–6735.

[bb48] Zhao, J., Li, S., Chen, S., Bai, Y. & Hu, J. (2012). *J. Coord. Chem.* **65**, 1201–1211.

[bb49] Zhu, X.-W., Xiao, B., Yin, Z.-G., Qian, H.-Y. & Li, G.-S. (2009). *Acta Cryst.* E**65**, m912. 10.1107/S1600536809026221 PMC2977252 21583370

